# Versatile *in situ* synthesis of MnO_2_ nanolayers on upconversion nanoparticles and their application in activatable fluorescence and MRI imaging†
†Electronic supplementary information (ESI) available. See DOI: 10.1039/c8sc00490k


**DOI:** 10.1039/c8sc00490k

**Published:** 2018-05-17

**Authors:** Yuan Wu, Dan Li, Fang Zhou, Hao Liang, Yuan Liu, Weijia Hou, Quan Yuan, Xiaobing Zhang, Weihong Tan

**Affiliations:** a Molecular Science and Biomedicine Laboratory , State Key Laboratory of Chemo/Bio-Sensing and Chemometrics , College of Biology , College of Chemistry and Chemical Engineering , Hunan University , Changsha , 410082 , China . Email: xbzhang@hnu.edu.cn ; Email: tan@chem.ufl.edu; b Institute of Molecular Medicine , Renji Hospital , Shanghai Jiao Tong University School of Medicine , College of Chemistry and Chemical Engineering , Shanghai Jiao Tong University , Shanghai 200240 , China; c Center for Research at Bio/Nano Interface , Department of Chemistry , Department of Physiology and Functional Genomics , Health Cancer Center , UF Genetics Institute and McKnight Brain Institute , University of Florida , Gainesville , Florida 32611-7200 , USA

## Abstract

We have developed a simple and versatile strategy for *in situ* growth of MnO_2_ on the surfaces of oleic acid-capped hydrophobic upconversion nanoparticles (UCNPs) by optimizing the component concentrations in the Lemieux–von Rudloff reagent. The oxidation time was shortened by a factor of two compared to that of the reported method. This oxidation process has no obvious adverse effects on the phases of UCNPs. STEM, X-ray photoelectron spectroscopy (XPS), Fourier transform infrared (FTIR) and energy-dispersive X-ray analysis (EDX) characterization demonstrated the successful growth of MnO_2_ on the surfaces of UCNPs. Furthermore, when the weight ratio of MnO_2_/UCNPs reached (147.61 ± 17.63) μg mg^–1^, 50% of the initial upconversion luminescence of UCNPs was quenched, as revealed by fluorescence and inductively coupled plasma optical emission spectrometry (ICP-OES) results. The presence of the surface MnO_2_ precipitate not only confers high dispersity of UCNPs in water, but also allows further activatable magnetic resonance imaging (MRI) and fluorescence multimodal imaging after reduction to Mn^2+^ by intracellular glutathione (GSH). A novel targeted drug carrier nanosystem was prepared to protect MnO_2_ from early decomposition in blood circulation by coating with mesoporous silica and capping with a gelatin nanolayer. Aptamer sgc8 was then attached to the surface of the gelatin nanolayer by covalent crosslinking to achieve targeted drug delivery. The results suggest that this nanosystem shows promise for further applications in cancer cell imaging and therapy.

## Abstract 2

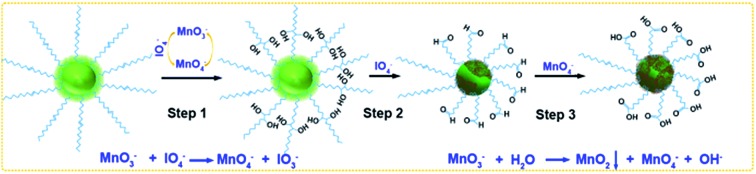
We have developed a simple and versatile strategy for *in situ* growth of MnO_2_ on the surfaces of oleic acid-capped upconversion nanoparticles by optimizing the component concentrations in the Lemieux–von Rudloff reagent.

## Introduction

Monodisperse nanocrystals with narrow size distribution synthesized by high-temperature thermolysis with oleic acid (OA) as the coordinating ligand show attractive features, including simple reactions without a further size-sorting process, tunable size *via* varying the ratio between OA and precursors, few crystalline defects, environmental friendliness and economical synthesis.^1^ Different kinds of OA-capped monodisperse nanocrystals have been extensively applied in biomedical applications, such as magnetic nanocrystals for magnetic resonance imaging (MRI), cargo carriers for drug-delivery, biosensors, and bioseparation,^2^–^9^ as well as semiconducting nanocrystals^10^–^14^ and UCNPs applied as fluorescent probes for cell tracking, biosensors, and cellular imaging.^15^–^20^

Because OA-capped monodisperse UCNPs are synthesized in organic media, their transfer to the aqueous phase and their functionalization are challenging. To solve these problems, many researchers have focused on transferring hydrophobic nanoparticles to the aqueous phase by ligand exchange, silanization, or hydrophobic–hydrophobic interactions.^21^ The oxidative cleavage of carbon–carbon double bonds to produce carboxylate groups using a catalytic amount of permanganate (KMnO_4_) in the presence of periodate (NaIO_4_) (Lemieux–von Rudloff reagent) was first reported in the mid-twentieth century.^22^ Li and coworkers used the Lemieux–von Rudloff reagent to oxidize OA ligands on the surfaces of UCNPs for dispersal in water.^23^ Subsequently, Liu and coworkers used the Lemieux–von Rudloff reagent to prepare hydrophilic UCNPs. Then 2-(*N*-morpholino)ethanesulfonic acid (MES) was used to reduce KMnO_4_ at pH 6.0 to form MnO_2_ nanosheets on the surfaces of hydrophilic UCNPs to serve as a quencher for upconverted luminescence.^24^ However, this oxidation process required two steps to form MnO_2_ and a total time of 48 h, and was both time-consuming and labor-intensive. To our knowledge, there have been no reports of MnO_2_ growth on the surfaces of UCNPs using the Lemieux–von Rudloff reagent to achieve oxidation and MnO_2_ formation of UCNPs in one pot.

**Scheme 1:**
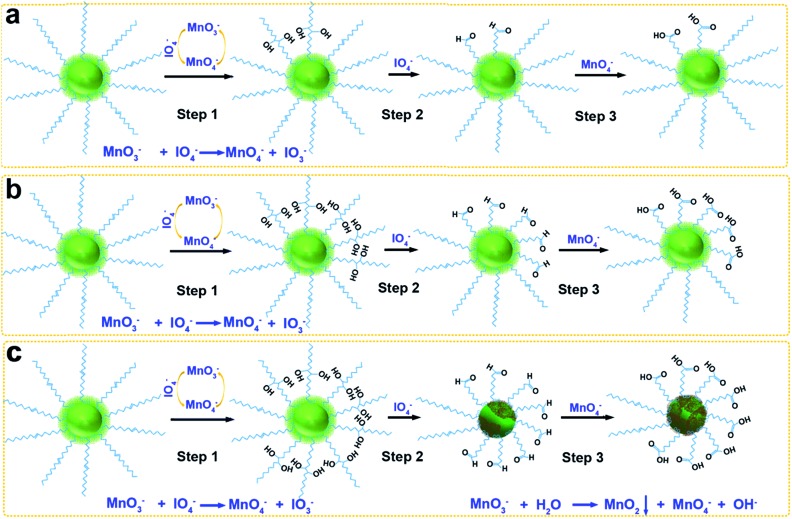
Synthesis mechanisms of MnO_2_-coated UCNPs from OA-capped precursors. (a) Control group 1: the molar ratio of OA : KMnO_4_ : NaIO_4_ was 1 : 3 : 56.5, as that in the reported method. (b) Control group 2: the molar ratio of OA : KMnO_4_ : NaIO_4_ was 1 : 9 : 56.5. (c) Experimental group: the molar ratio of OA : KMnO_4_ : NaIO_4_ was 1 : 9 : 18.8.

To solve these obstacles, we have optimized the concentrations of KMnO_4_ and NaIO_4_ in a mildly alkaline solution (pH 7.7) to accelerate the oxidation of OA, and this oxidation was accompanied by the growth of MnO_2_ on the surfaces of UCNPs (Scheme 1c). Two control experiments were carried out as described in the ESI† (Scheme 1a and b).

Moreover, our group has reported the studies on using simple MnO_2_ nanosheets to achieve gene-silencing therapy, cellular imaging and photodynamic therapy *via* reducing glutathione levels in cancer cells.^25^,^26^ In these reports, MnO_2_ nanosheets were intratumorally injected into athymic nude mice. Glutathione (GSH) molecules participate in many physiological processes not only in the cells, but also in blood with a concentration range from 0.8 to 15 mM.^27^ MnO_2_ nanosheets can be converted to Mn^2+^*via* GSH reduction, but it is impossible for MnO_2_ to differentiate between the intracellular GSH and extracellular GSH *in vivo*. Hypothetically, MnO_2_ nanosheets were intravenously injected in *in vivo* experiments, MnO_2_ nanosheets may be reduced partly by GSH in blood, which would lead to false signal imaging or early cargo release before arriving at the tumor, decreased therapeutic efficiency, and side effects. So it is considerably necessary to protect MnO_2_ in the blood circulation, and to promise that MnO_2_ decomposition only happens in target cells or tissues. In the present work, a novel targeted drug carrier nanosystem was successfully fabricated to protect MnO_2_ from early decomposition in blood circulation by coating with mesoporous silica and capping with a gelatin nanolayer. More detailed information about this nanosystem is in the application part.

## Results and discussion

### Preparation and characterization of UCNPs@MnO_2_

**Fig. 1:**
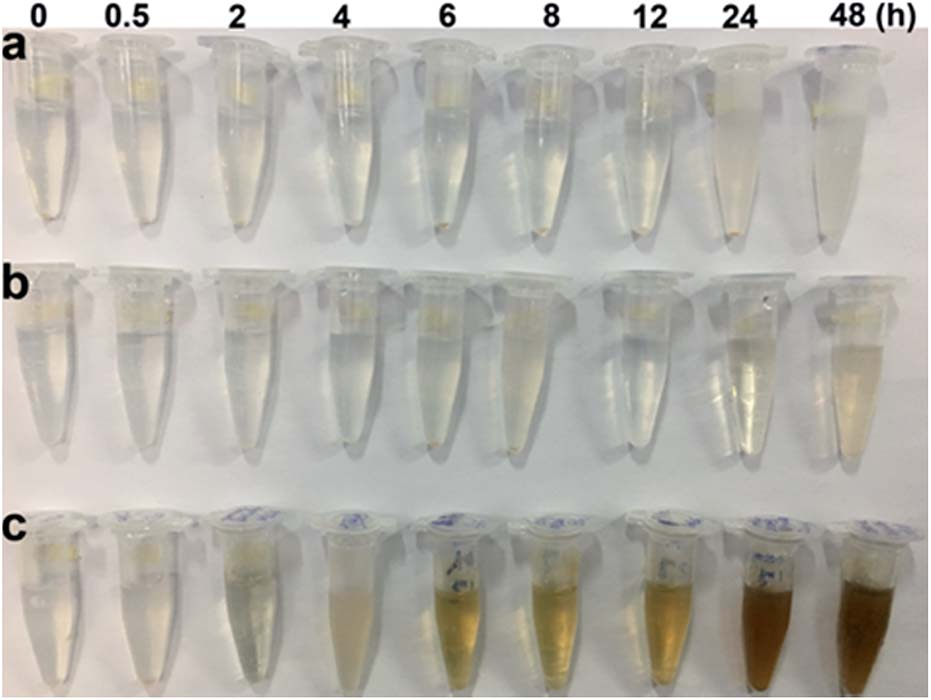
Pictures of the products isolated and dispersed in PBS (1 mg mL^–1^) after different oxidation times. (a) Control group 1 (the concentration of KMnO_4_ and NaIO_4_ was 0.556 mM and 10.244 mM, respectively, as those in the reported method.); (b) control group 2 (the concentration of KMnO_4_ and NaIO_4_ was 1.668 mM and 10.244 mM, respectively); (c) experiment group (the concentration of KMnO_4_ and NaIO_4_ was 1.668 and 3.415 mM, respectively).

**Fig. 2:**
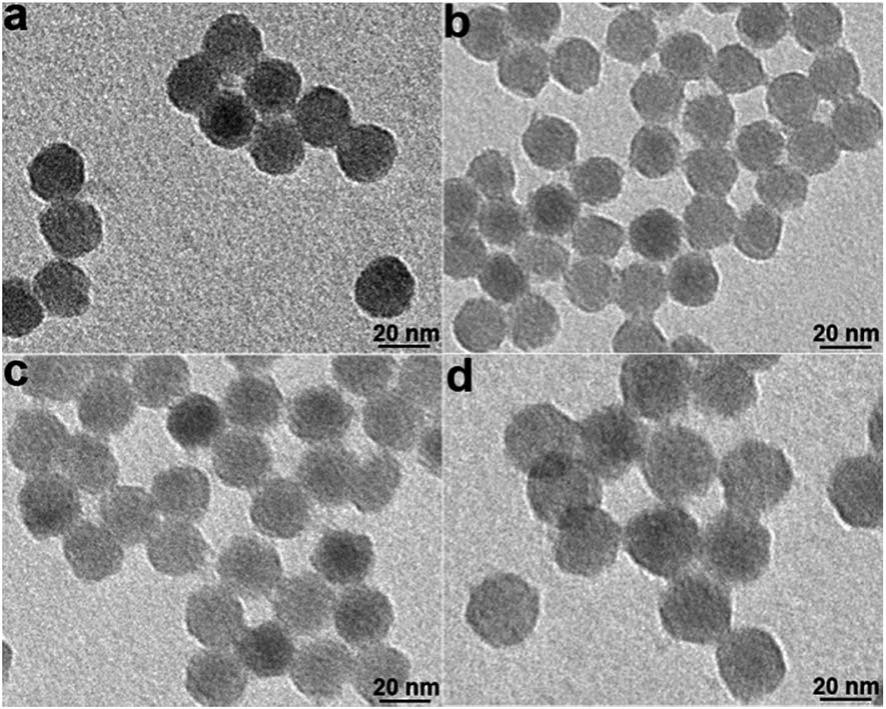
TEM images of the oxidized UCNPs from the experimental group at different times. (a) 2 h; (b) 6 h; (c) 12 h; (d) 24 h.

NaYF_4_:Yb/Gd/Er UCNPs were synthesized according to a reported procedure.^28^ Transmission electron microscopy (TEM) imaging of UCNPs demonstrated their monodisperse particle size of about 22 nm (Fig. S1†). The Lemieux–von Rudloff reagent was used to oxidize the as-prepared OA-capped UCNPs. During the course of our investigation using the Lemieux–von Rudloff reagent, we discovered that the typical pink color of KMnO_4_ changed (Fig. S2a†) after 60 h, and 74.6% of the upconverted luminescence of the obtained dark-brown UCNPs was quenched (Fig. S2b†). The fluorescence recovered after adding GSH. On the basis of these findings, we hypothesized that this oxidation reaction was accompanied by precipitation of MnO_2_ on the surfaces of UCNPs. However, the reaction time was rather long using the reported procedure. Therefore, we optimized the concentrations of KMnO_4_ and NaIO_4_ in the Lemieux–von Rudloff reagent (a two-fold higher concentration of KMnO_4_ and a two-fold lower concentration of NaIO_4_ than those of the reported method were used). Pictures of the reaction mixture at different times are shown in Fig. S3.† The products isolated after different reaction times in the course of the oxidation of OA-capped UCNPs are shown in Fig. 1. The color change due to product formation is clearly visible, and the dispersibility of UCNPs was much better after 6 h (Fig. 1c) under the optimized conditions compared to those of the two control groups. TEM images (Fig. 2) of products isolated after different times from the experimental group clearly showed the precipitate formed on the surface of UCNPs compared to Fig. S1† of OA-capped UCNPs.

**Fig. 3:**
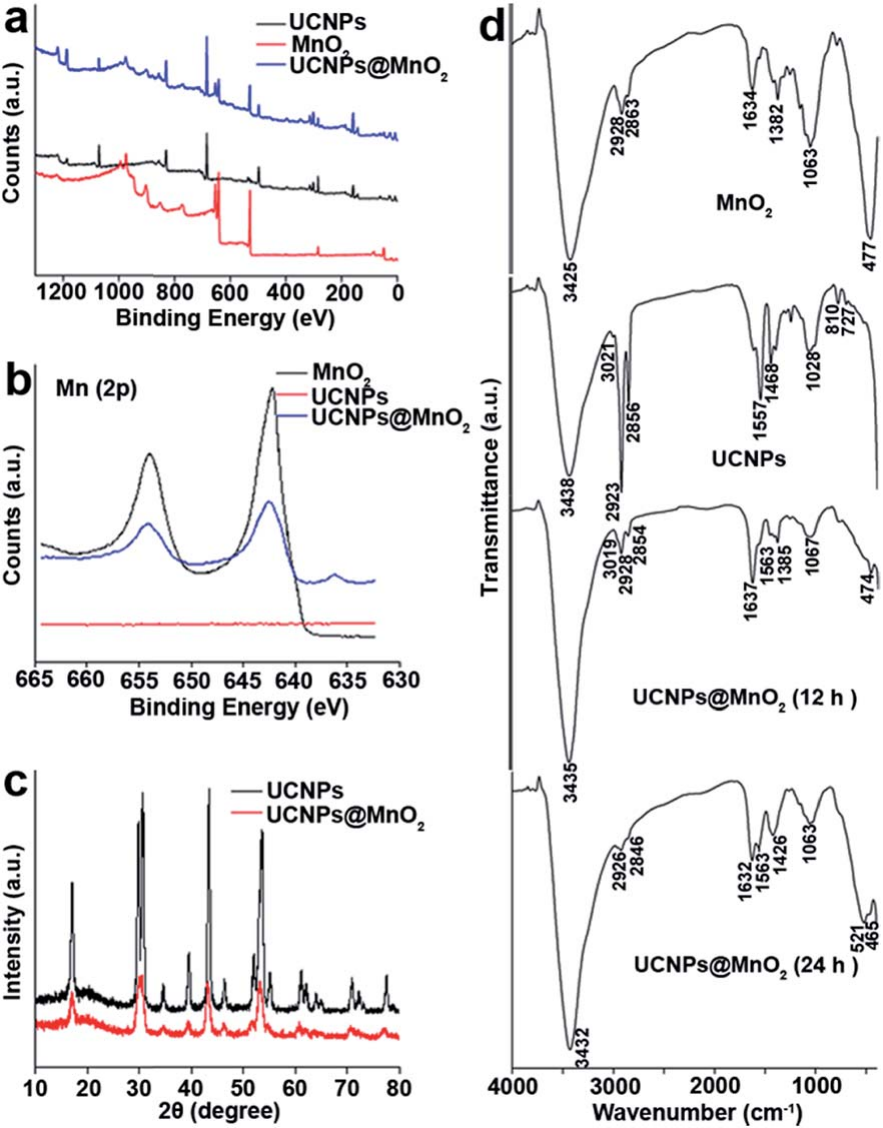
(a) XPS spectra of UCNPs, MnO_2_ and UCNPs@MnO_2_. (b) High resolution Mn (2p) XPS spectra of UCNPs, MnO_2_ and UCNPs@MnO_2_. (c) XRD spectra of UCNPs and UCNPs@MnO_2_. (d) FT-IR spectra of MnO_2_, UCNPs, and UCNPs@MnO_2_ at different oxidation times (12 h and 24 h).

The identity of the precipitate formed on the surfaces of UCNPs was determined by energy-dispersive X-ray spectroscopy (EDX), scanning transmission electron microscopy (STEM), and X-ray photoelectron spectroscopy (XPS). Compositional analysis by EDX indicated the presence of a new element (Mn) not observed in the EDX data for UCNPs (Fig. S4†). The existence of Mn on the surfaces of UCNPs was proved by elemental mapping with STEM (Fig. S5†). Ultrathin MnO_2_ nanosheets were prepared as a positive control according to a reported method,^29^ and the characterization is shown in Fig. S6.† According to the XPS analysis (Fig. 3a and b), the peaks of Mn 2p_3/2_ at 642.2 eV and 2p_1/2_ at 653.8 eV further indicate the presence of MnO_2_.^30^,^31^ From the EDX, STEM and XPS results, it can be confirmed that the precipitate formed on the surfaces of UCNPs was MnO_2_. XRD patterns of both the as-prepared and oxidized UCNPs were studied to investigate the phase effect of the oxidation process. From the XRD result shown in Fig. 3c, it can be concluded that the oxidation had no obvious adverse effect on the phase of UCNPs.

The capping ligands on the surfaces of UCNPs were identified by FTIR spectra in Fig. 3d. The MnO_2_ nanosheets, the as-prepared UCNPs and oxidized UCNPs at different reaction times all exhibit a broad band at around 3430 cm^–1^, corresponding to the O–H stretching vibration. A peak at 3020 cm^–1^ attributed to the =C–H stretching vibration can be observed in the spectrum of UCNPs and UCNPs@MnO_2_ (12 h),^32^ but this feature is apparently lost in the spectrum of UCNPs@MnO_2_ (24 h), suggesting that all of the OA ligands on the surfaces of UCNPs were oxidized to azelaic acid ligands after 24 h. In addition, bands at 1557 and 1468 cm^–1^ observed in the spectrum of the UCNPs are attributed to the asymmetric and symmetric stretching vibrations of the carboxylate group of the OA ligand. However, in the two cases of the oxidized samples, bands corresponding to the carboxylate group are found at 1637 and 1563 cm^–1^, and 1632 and 1563 cm^–1^, respectively. The obvious changes of bands at around 810 cm^–1^ and 727 cm^–1^ observed in the spectrum of the as-prepared UCNPs and the oxidized UCNPs samples are associated with the external deformation vibration of =C–H, which decreases in intensity with oxidation time. These results suggested the cleavage of the –HC=CH– group of the bound OA. The FT-IR spectrum also showed the characteristic absorbance of the Mn–O stretching vibration at around 470 cm^–1^ in the spectrum of MnO_2_ and both of the oxidized samples,^33^,^34^ indicating the existence of MnO_2_ on the surfaces of the oxidized UCNPs. On the basis of the above FTIR results, it can be deduced that the OA ligands on the surfaces of UCNPs were oxidized to azelaic acids, and the oxidation process was accompanied by the successful growth of MnO_2_ on the surfaces of UCNPs.

To evaluate the ligand content in the as-prepared UCNPs and oxidized UCNPs@MnO_2_ samples, thermogravimetric analysis (TGA) was performed (Fig. S7†). The oxidized UCNPs@MnO_2_ samples were treated with GSH to remove the effect of the surface MnO_2_. The weight loss in the temperature range of 30–200 °C is due to the loss of adsorbed water from each sample, and a further weight loss observed from 200 to 460 °C is attributed to the combustion of the organic groups on the surfaces of UCNPs in the samples, and reflects the ligand content. The content of OA ligands in the UCNPs was about 10.5% (Fig. S7†), so we could calculate the amount of OA ligands on the surfaces of UCNPs. The ligand content of oxidized UCNPs@MnO_2_ (24 h) decreased to 7.2%, which was consistent with the calculated result (see the ESI†) according to the molecular weight of ligands (282.46 for OA and 188.22 for azelaic acid). Therefore, it could be concluded that almost all of the OA ligands on the surfaces of UCNPs had been oxidized to azelaic acid after 24 h. The ligand content of oxidized UCNPs@MnO_2_ (12 h) decreased to 8.7%, which was between 7.2% and 10.5%, indicating that 56.2% of OA ligands were oxidized to azelaic acid, which was consistent with the FTIR result.

To investigate the mechanism of MnO_2_ growth on the surfaces of UCNPs, control group 3 (see the ESI†) was prepared in the absence of OA-capped-UCNPs. The results showed that no changes in the color of the reaction mixture occurred after one week, and no new product was produced after centrifugation (Fig. S8†), thus indicating that the KMnO_4_ and NaIO_4_ solutions by themselves are quite stable under the experimental conditions, and further indicating the specificity towards olefinic bonds of this oxidation reagent. Thus, the reagents alone could not bring about the growth of MnO_2_. In the experimental group, the supernatants after centrifugation were collected after the oxidation reaction (Fig. S9†). It was observed that the colors of all the supernatants changed after 3 days, which means that the MnO_2_ precipitate came from the reaction of intermediates formed during the oxidation process.

**Fig. 4:**
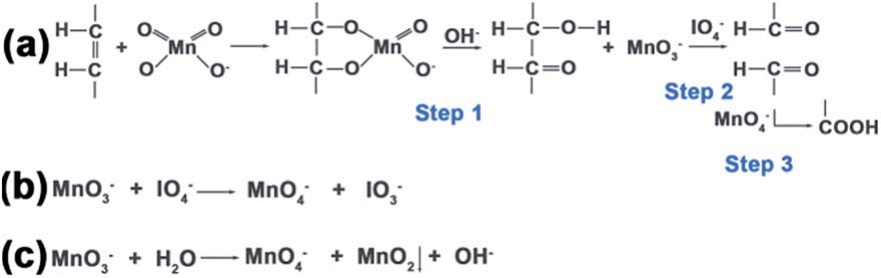
Scheme for the oxidation reactions. (a) The periodate-permanganate oxidation process of carbon–carbon double bonds can be explained in three steps. (b) The reaction of the IO_4_^–^ to regenerate MnO_4_^–^ from its reduced state of MnO_3_^–^. (c) The disproportionation reaction of MnO_3_^–^ to form the MnO_2_ precipitate.

The oxidation process can be explained in three steps (Fig. 4a): first MnO_4_^–^ oxidation of carbon–carbon double bonds to cyclic permanganate esters, followed by hydrolysis to hydroxyketones (step 1), then the rapid IO_4_^–^ cleavage of the hydroxyketone products (step 2), and finally MnO_4_^–^ oxidation of the second stage products to carboxyl groups (step 3). In control group 1 (Scheme 1a), the molar ratio of OA : KMnO_4_ : NaIO_4_ was 1 : 3 : 56.5. The amount of KMnO_4_ was considerably smaller than that of NaIO_4_ due to the ability of the IO_4_^–^ to regenerate MnO_4_^–^ from its reduced state (Fig. 4b). Because the reaction occurred in a heterogeneous reaction system in a mildly alkaline solution (pH 7.7), the reaction time was rather long. Moreover, no redundant MnO_3_^–^ could form the MnO_2_ precipitate. In control group 2 (Scheme 1b), the molar ratio of OA : KMnO_4_ : NaIO_4_ was 1 : 9 : 56.5. Only the concentration of KMnO_4_ was increased, the amount of NaIO_4_ was still considerably larger than that of KMnO_4_. The MnO_3_^–^ was oxidized to MnO_4_^–^ by IO_4_^–^ once MnO_3_^–^ was produced, so the initial step 1 was faster, but few MnO_2_ precipitates were formed. In the experimental group (Scheme 1c), the molar ratio of OA : KMnO_4_ : NaIO_4_ was 1 : 9 : 18.8. The concentration of permanganate was increased, increasing the rate of oxidation in step 1, and accelerating the entire oxidation process. At the same time, the concentration of periodate was decreased. Thus, the probability for IO_4_^–^ to oxidize MnO_3_^–^ to MnO_4_^–^ decreased, and finally the MnO_2_ precipitate was formed by the disproportionation reaction of MnO_3_^–^ (Fig. 4c).^35^,^36^ Since IO_4_^–^ does not oxidize MnO_2_ in alkaline media, the oxidized UCNPs acted as templates to aid the growth of the MnO_2_ precipitate in this heterogeneous reaction system. Further work on the more detailed mechanism and effects of pH and temperature on this reaction is now in progress.

As a result of the ligand change from OA to azelaic acid and the growth of MnO_2_ on the surface of UCNPs, the results presented in Fig. 1 and S11† show the dispersibility of the products formed at different times, and we can conclude that the growth of MnO_2_ on the surfaces of UCNPs improved the dispersibility of UCNPs. When MnO_2_ was reduced to Mn^2+^ with GSH, the dispersibility (Fig. S10 and S12†) was improved considerably in our experimental group after 18 h. However, the dispersibility of the two control groups was poor because of the incomplete oxidation of OA ligands. Again, these results indicate that our method increases the rate of the entire oxidation process.

**Fig. 5:**
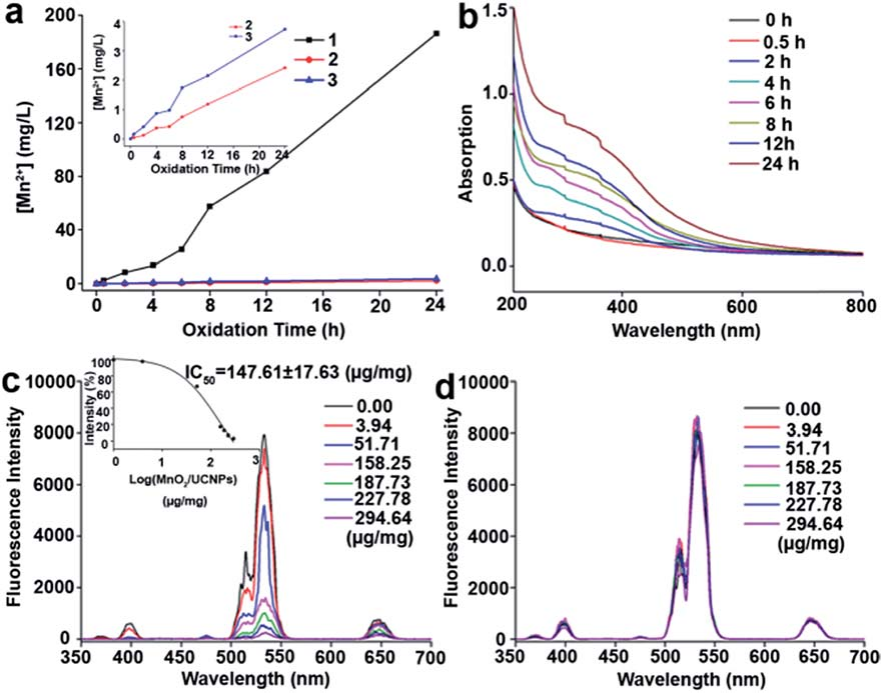
(a) ICP-OES results of the oxidized UCNPs at different oxidation times. (1) Experimental group, (2) control group 1, and (3) control group 2. (Inset) Enlarged view of the ICP-OES results of control groups 1 and 2. (b) UV-vis absorption spectra of the oxidized UCNPs at different oxidation times in the experimental group. (c) Fluorescence response of the oxidized UCNPs at different MnO_2_/UCNP concentration ratios (0–294.64 μg mg^–1^). (Inset) Plot of normalized fluorescence intensity (%) at 533 nm *versus* the log[MnO_2_/UCNPs]. The spectra were recorded by excitation with a 980 nm laser at a current density of 2 W cm^–2^. (d) Fluorescence recovery response of the samples in (c) after adding 10 mM GSH.

The amounts of MnO_2_ formed on the surfaces of UCNPs at different oxidation times were determined by ICP-OES (Fig. 5a). The UV-vis absorption spectra in Fig. 5b and S13† show a characteristic broad peak centered at 380 nm for MnO_2_ nanomaterials, which is consistent with the previous reports.^25^,^28^ From ICP-OES and UV results, it was clear that the amounts of MnO_2_ increase with oxidation time using the optimized composition of the Lemieux–von Rudloff reagent.

The luminescence properties of the oxidized samples in aqueous solution presented in Fig. 5c and S14a† confirmed the quenching ability of MnO_2_ on the luminescence of UCNPs due to energy transfer between UCNPs and MnO_2_.^24^ When the weight ratio of MnO_2_ on the UCNPs reached about (147.61 ± 17.63) μg mg^–1^, 50% of the initial upconversion luminescence of UCNPs was quenched. The luminescence of UCNPs was recovered in the presence of GSH (Fig. 5d and S14b†).

### Applications

Most importantly, the presence of MnO_2_ on the surfaces of UCNPs facilitates further biological applications. Typically, MnO_2_ nanosheets can be converted to Mn^2+^*via* intracellular GSH reduction, leading to activatable MRI.^37^ Moreover, MnO_2_ can quench the upconversion luminescence of UCNPs, leading to potentially activatable fluorescence imaging. But to our knowledge, no strategy has been reported to protect MnO_2_ nanosheets from early reduction by GSH in blood circulation, which would lead to false signal imaging or early cargo release.

A novel targeted drug carrier nanosystem (Scheme S1†) was successfully fabricated to protect MnO_2_ from early decomposition in blood circulation by coating with mesoporous silica and capping with a gelatin nanolayer. First, we followed the reported method to obtain mesoporous silica-coated UCNPs@MnO_2_ (UCNPs@MnO_2_@mSiO_2_) characterized by TEM, EDX and FT-IR (Fig. S15†).^38^ Then the as-prepared UCNPs@MnO_2_@mSiO_2_ was capped with gelatin (UCNPs@MnO_2_@mSiO_2_@gel nanosystem) following the reported method,^39^ as confirmed by TEM (Fig. S16a†). Finally, sgc8 aptamers, bind to the cell membrane protein PTK7 with high affinity and selectivity (*K*_d_ = 0.8 ± 0.09 nM),^40^ were conjugated on the gelatin surface (sgc8-nanosystem is short for UCNPs@MnO_2_@mSiO_2_@gel-sgc8) through the bifunctional cross-linker sulfosuccinimidyl 4-[*N*-maleimidomethyl] cyclohexane-1-carboxylate (sulfo-SMCC)^41^ to achieve specific affinity. Zeta potential tests (Fig. S16b†) and agarose gel electrophoresis tests (Fig. S16c†) confirmed the successful conjugation of sgc8 aptamers on the gelatin surface.

**Fig. 6:**
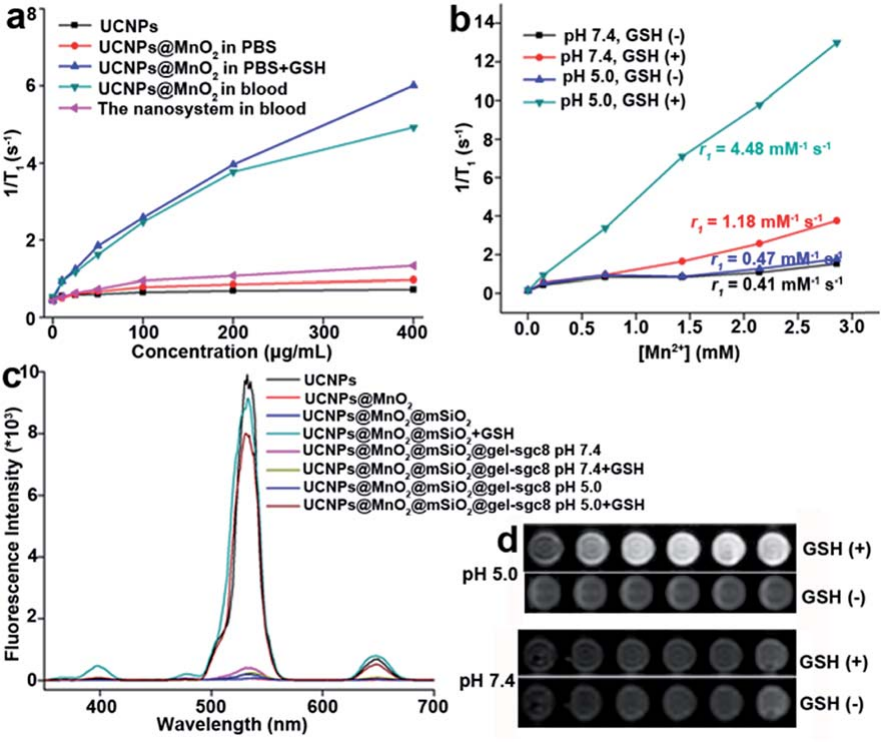
(a) Plot of 1/*T*_1_*versus* concentration of different nanoparticles (0, 10, 25, 50, 100, 200, and 400 μg mL^–1^) in blood and in PBS. (b) Plots of 1/*T*_1_*versus* [Mn^2+^] for the sgc8-nanosystem with or without GSH at pH 7.4 and pH 5.0. (c) Fluorescence quenching and recovery of different synthesized nanoparticles at pH 5.0 or pH 7.4, with or without GSH. (d) *T*_1_-Weighted MRI results of the sgc8-nanosystem at pH 5.0 (top) and pH 7.4 (bottom). Left to right: [Mn^2+^] = 0.00, 0.14, 0.71, 1.42, 2.14, 2.85 mM.

The concentration of GSH in human whole blood is reported to be 849 ± 63 μM.^42^,^43^ To confirm the necessity to protect MnO_2_ from early decomposition, we tested the longitudinal relaxation rate (1/*T*_1_) of UCNPs@MnO_2_ and the gelatin-protected nanosystem when exposed to normal human whole blood. The results (Fig. 6a) showed that UCNPs@MnO_2_ in blood does have a higher longitudinal relaxation rate than that in PBS or in the gelatin-protected nanosystem in blood. This demonstrates the necessity to protect MnO_2_ from early reduction in blood when being applied in biomedical research, and confirms that the gelatin nanolayer indeed can protect MnO_2_ from early reduction in blood. We next tested the longitudinal relaxation rate (1/*T*_1_) (Fig. 6b) and MRI (Fig. 6d) of the as-prepared gelatin-protected nanosystem at different pH values with or without GSH *in vitro*. These results suggested that our nanosystem can provide acid- and GSH-activated magnetic resonance signals for MRI. The fluorescence properties of the as-prepared nanosystem tested in Fig. 6c and S17† showed that the fluorescence intensity of UCNPs within the nanosystem could be efficiently quenched and could be recovered only when the environment was acidic with GSH, thereby indicating the potential for application of our nanosystem in activatable fluorescence imaging, and confirming the ability of the gelatin nanolayer to protect the inner MnO_2_ nanolayer from early decomposition in the physiological environment.

This protecting ability can be reversed by the acid-responsive properties of gelatin. After crosslinking, at neutral pH, the charge of the cross-linked gelatin was positive (4.7 mV) (Fig. S18†), and the charge of mesoporous silica was negative (–11.8 mV). The electrostatic attraction force between the gelatin coating layer and mesoporous silica could stabilize the pore-blocking capability of the gelatin layer. However, the charge of the cross-linked gelatin layer was negative (–10.4 mV) in the acidic environment (pH 5.0). Hence, the increased electrostatic repulsive force between the gelatin coating layer and mesoporous silica could open the pore and allow the escape of the drug and the entrapped GSH molecules.^44^,^45^ Subsequently, the MnO_2_ nanolayer is reduced to Mn^2+^ ions by intracellular GSH, which, in turn, facilitates the fluorescence recovery of UCNPs. Meanwhile, Mn^2+^ ions can act as the contrast agent for MRI, giving an activatable upconverted fluorescence signal and magnetic resonance signal.

**Fig. 7:**
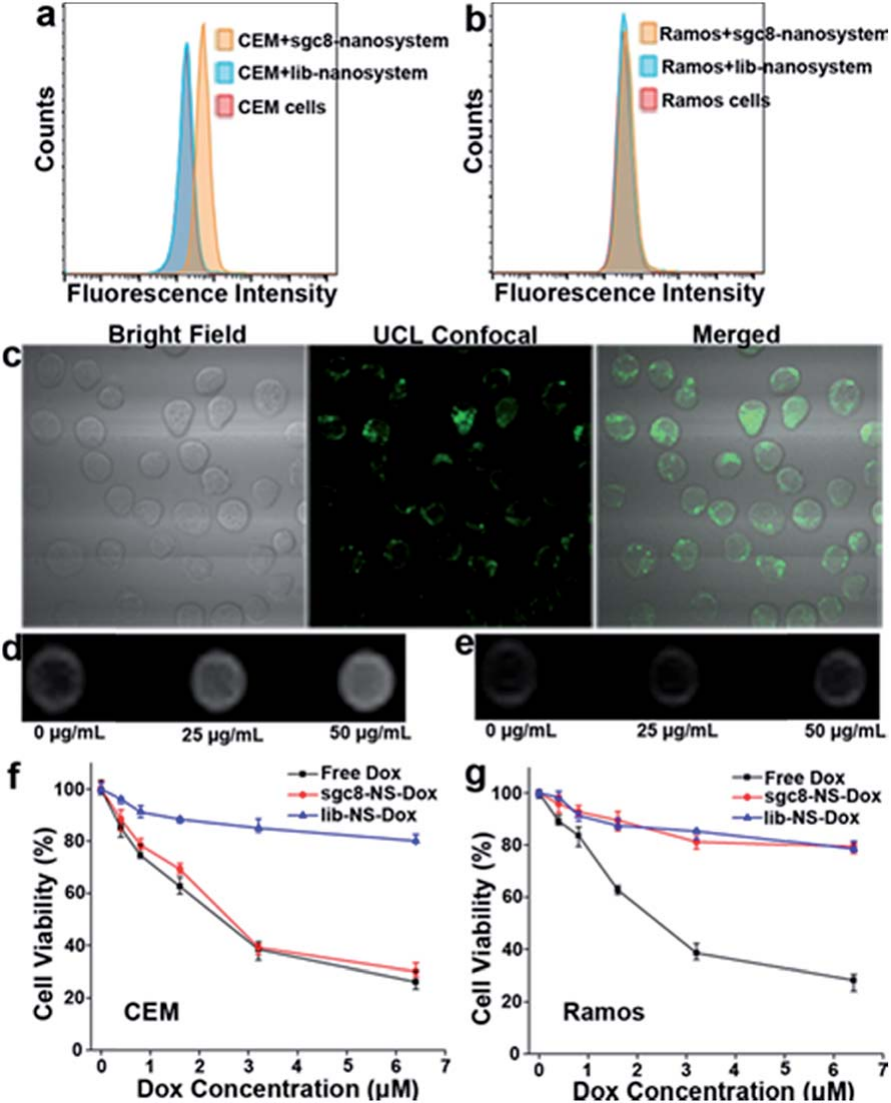
Biomedical applications of the multifunctional nanosystem for selective cancer cell recognition, intracellular bioimaging, and targeted anticancer drug delivery. Flow cytometry results of (a) target CEM cells and (b) nontarget Ramos cells. (c) Confocal microscopy images showing that the sgc8-modified nanosystem was internalized into target CEM cells after incubation at 37 °C for 4 h, and the fluorescence of UCNPs was recovered. *T*_1_-Weighted MRI images of (d) CEM cells and (e) Ramos cells treated with the sgc8-nanosystem at concentrations of 0, 25, and 50 μg mL^–1^. Cytotoxicity of the Dox-loaded sgc8-nanosystem (sgc8-NS-Dox) and the lib-nanosystem (lib-NS-Dox) in (f) CEM cells and (g) Ramos cells, in contrast to nonselective toxicity of free Dox in both cell lines.

CCRF-CEM cells with high membrane PTK7 expression were chosen as the target cancer cell line and Ramos cells without membrane PTK7 expression were used as a negative control cell line.^46^ The binding and internalization abilities of the sgc8-nanosystem toward targeted CEM cells and negative control Ramos cells were evaluated using flow cytometry analysis. A noticeably higher enhancement in the fluorescence signal was observed for CEM cells treated with the sgc8-nanosystem compared to CEM cells treated with the random sequence-modified nanosystem (lib-nanosystem) (Fig. 7a), while no significant change in fluorescence intensity was observed for negative Ramos cells (Fig. 7b). The selectivity was also confirmed using two-photon confocal laser scanning microscopy^47^ (Fig. 7c and S19†). To demonstrate activatable fluorescence imaging, HeLa cells with high PTK7 expression, another cell line targeted by sgc8 aptamers,^48^ were chosen to perform time scanning imaging as shown in Fig. S20,† indicating the “off–on” imaging mode of our sgc8-nanosystem due to the time-dependent dissolution of the gelatin-coated layer. These results provide powerful support for the mechanism of target cell-activated fluorescence. Next, the feasibility of the sgc8-nanosystem for cellular MRI was investigated by treatment of CEM and Ramos cells with different concentrations of the sgc8-nanosystem. It can be seen in Fig. 7d and e that CEM cells treated with the sgc8-nanosystem presented enhanced *T*_1_-weighted MRI images compared to CEM cells alone and Ramos cells treated with the sgc8-nanosystem. The amounts of Mn^2+^ in each CEM cell and Ramos cell treated with 50 μg mL^–1^ sgc8-nanosystem were 0.073 and 0.009 pg, respectively, tested by ICP-OES. These results demonstrated that our designed nanosystem can be used as a luminescent probe and as an MRI contrast agent for live cell imaging. As a proof-of-delivery concept, Dox was loaded into the nanosystem (sgc8-nanosystem-Dox). To investigate the acid-induced and controlled-release properties, the sgc8-nanosystem-Dox was exposed to buffers with different pH values at 4.0, 5.0, 6.0 and 7.4, as shown in Fig. S21.† The colocalization study of the sgc8-nanosystem with LysoTracker Green in HeLa cells is shown in Fig. S22.† The results revealed that the release rate of this pH-responsive sgc8-nanosystem-Dox increased over a 5 h period in mimicked environments of late endosome and lysosome, where the pH values would be in the range of 5.0–6.0.^49^ Finally, cell viability tests (Fig. 7f and g and S23†) were performed to confirm that the aptamer-modified drug delivery system would facilitate the target cell killing activity of the drug. In contrast to nonselective cytotoxicity of free Dox in both target and nontarget cells, selective cytotoxicity induced by the sgc8-modified nanosystem in target cells demonstrated the applicability of our aptamer-modified nanosystem for targeted drug delivery.

## Conclusion

In conclusion, by optimizing the component concentrations in the Lemieux–von Rudloff reagent, we have broadened the application of this reagent in transferring OA-capped UCNPs into an aqueous phase, to include the direct formation of a MnO_2_ nanolayer on the surfaces of the oxidized UCNPs. The oxidation time was shortened to half the time of the reported method. Detailed investigations confirmed the existence of the MnO_2_ precipitate and provided information about the degree of oxidation at different oxidation times. It should be noted that our finding on the color change when using the Lemieux–von Rudloff reagent can be used as an indicator to track the oxidation process. Moreover, we believe that this MnO_2_ growth method is not limited to hydrophobic UCNPs. It can be a common strategy applied to other hydrophobic nanoparticles synthesized by high-temperature thermolysis, such as semiconductor and metal nanoparticles, where only the coordinating ligands will be oxidized by the Lemieux–von Rudloff reagent. Coincidentally, the surface MnO_2_ precipitate can act as a common quencher of the fluorescence of the host nanoparticles. The fluorescence can be totally recovered by reducing agents such as GSH and DTT, indicating potential for activatable fluorescence imaging application.

Last, but also important, we provided a method to protect MnO_2_ from early reduction by GSH in blood by coating with an acid-responsive gelatin nanolayer for more accurate imaging signals. The successful growth of MnO_2_ on the surfaces of UCNPs and the fabrication of the targeted delivery and imaging system suggest that it is possible to expand the application of the Lemieux–von Rudloff reagent and these UCNPs@MnO_2_ carriers as activatable luminescent labels and contrast agents to other biological fields, such as bioimaging and cancer cell therapy.

## Conflicts of interest

There are no conflicts to declare.

## Supplementary Material

Supplementary information
