# Supplementary material for: Versatile in situ synthesis of MnO2 nanolayers on upconversion nanoparticles and their application in activatable fluorescence and MRI imaging
Source: Chem Sci. 2018 May 17;9(24):5427–34. doi: 10.1039/c8sc00490k (PMC6009534; doi:10.1039/c8sc00490k)
Supplement: Supplementary file 1 [file SC-009-C8SC00490K-s001.pdf]

Electronic Supplementary Material (ESI) for Chemical Science.
This journal is © The Royal Society of Chemistry 2018

Supporting Information

Versatile *in-situ* Synthesis of MnO_2 Nanolayers on Upconverting Nanoparticles and Application for Activatable Fluorescence and MRI Imaging

Yuan Wu,[†‡] Dan Li,[†] Fang Zhou,[†] Hao Liang,[†] Yuan Liu,[†‡] Weijia Hou,[‡] Quan Yuan,[†] Xiaobing Zhang,*[†] Weihong Tan*[†§‡]

[†] Molecular Science and Biomedicine Laboratory, State Key Laboratory of Chemo/Bio-Sensing and Chemometrics, College of Biology and College of Chemistry and Chemical Engineering, Hunan University, Changsha, 410082, China

[§] Institute of Molecular Medicine, Renji Hospital, Shanghai Jiao Tong University School of Medicine and College of Chemistry and Chemical Engineering, Shanghai Jiao Tong University, Shanghai 200240, China

[‡] Center for Research at Bio/Nano Interface, Department of Chemistry and Department of Physiology and Functional Genomics, Health Cancer Center, UF Genetics Institute and McKnight Brain Institute, University of Florida, Gainesville, Florida 32611-7200, United States

E-mail: tan@chem.ufl.edu xbzhang@hnu.edu.cn

Chemicals. Yttrium(III) acetate hydrate (99.9%), ytterbium(III) acetate hydrate (99.9%), erbium(III) acetate hydrate (99.9%), sodium hydroxide (NaOH; >98%), ammonium fluoride (NH4F; >98%), 1-octadecene (90%), oleic acid (90%), Oleic acid (OA), 1-octadecene (ODE), N-Cetyltrimethylammonium chloride (CTAC), *tert*-butanol, potassium carbonate(K_2CO_3), sodium periodate ($NaIO_4$), potassium permanganate ($KMnO_4$), and tetraethylorthosilicate (TEOS, 28%) were obtained from Alfa Aesar. Manganese chloride tetrahydrate ($MnCl_2$•$4H_2O$), tetramethylammonium hydroxide, hydrogen peroxide (H_2O_2, 30 wt%), cowhide gelatin, and glutaraldehyde were purchased from Sigma-Aldrich and used as received without further purification. RPMI-1640 medium, penicillin streptomycin solution and fetal bovine serum were

obtained from Invitrogen. Dox was purchased from Frontier Scientific. Cell Titer 96 Aqueous One Solution cell proliferation assay (MTS) was purchased from Promega. All other chemicals were of analytical grade, purchased from Sinopharm Chemical Reagent Co., Ltd. (Shanghai, China), and used without further purification. Ultrapure Milli-Q water (Millipore) was used throughout the experiments.

Normal whole human blood. Normal whole human blood was collected by Xiangya Hospital (Changsha, China) from a volunteer, and prepared using standard protocols.[1] For whole blood experiments, blood was collected in heparin-coated vacutainers (BD, Fisher Scientific) to reduce clotting. Whole blood samples were stored at 4 °C for experiments in one week. All whole blood experiments were performed in compliance with the relevant laws and guidelines from Xiangya Hospital, and the committee of Xiangya Hospital has approved the research experiments. The volunteer has also approved that the blood can be used for research experiments.

Cell lines. Human acute lymphoblastic leukemia CCRF-CEM cell line, human Burkitt's lymphoma Ramos cell line and HeLa cells were cultured in RPMI 1640 medium with 10% fetal bovine serum (FBS) and 0.5 mg/mL penicillin streptomycin at 37 °C under a 5% CO_2 atmosphere. The cell lines were bought from American type culture collection (ATCC), and the cell lines were cultured according to the guidelines of ATCC. Cells were washed before and after incubation with washing buffer. Cells were washed before and after incubation with washing buffer [4.5 g/L glucose and 5 mM $MgCl_2$ in Dulbecco's PBS with calcium chloride and magnesium chloride (Sigma-Aldrich)]. Binding buffer used for the binding test was prepared by adding yeast tRNA (0.1 mg/mL; Sigma-Aldrich) and BSA (1 mg/mL; Fisher Scientific) to the washing buffer to reduce background binding.

Instruments.

Zeta potential was measured on the Malvern Zetasizer Nano ZS90 (Malvern Instruments, Ltd., Worcestershire, UK). Atomic force microscopy (**AFM**) images of samples were obtained on a Multimode 8 (Bruker, USA). Transmission electron microscopy (**TEM**) images and energy-dispersive X-ray analysis (EDX) data were

obtained on an H-7000 NAR transmission electron microscope (Hitachi) with a working voltage of 100 kV. Scanning transmission electron microscopy (STEM) imaging was performed by field emission electron microscopy (Titan G2 60-300, FEI company, USA). X-ray photoelectron spectroscopy (**XPS**) spectra were obtained on an ESCALAB 250Xi XPS system (ThermoFisher-VG Scientific) with a monochromatized Al Kα x-ray source at 12 kV and 6 mA. Powder X-ray diffraction (**XRD**) spectra were obtained on an XRD-6100 (Shimadzu). **FT-IR** spectra were obtained on a Nicolet iS10 FT-IR spectrometer (Thermo Scientific). Thermogravimetric analysis (**TGA**) was performed on an STA449C (Germany) at a heating rate of 10 °C/min under argon. Inductively coupled plasma optical emission spectrometry (**ICP-OES**) data were obtained on an Optima 8000 ICP-OES spectrometer (Perkin-Elmer). Flow cytometer measurements were obtained on a FACScan cytometer (Becton Dickinson). Fluorescence spectra were recorded on an F4500 spectrometer (Hitachi) in conjuction with a 980 nm diode laser. Fluorescence imaging was recorded on an Olympus FV 500-IX81 confocal microscope equipped with a commercial CW IR laser (980 nm). The MTS assay was obtained on a Multiskan GO UV/Vis microplate spectrophotometer (Thermo Scientific), and the absorbance value at 570 nm was determined by a VersaMax microplate reader (Molecular Devices, Inc.).

Synthesis of $NaYF_4$: 24%Gd/20%Yb/2%Er Nanoparticles. In a typical experiment, to a 50-mL flask containing oleic acid (3 mL) and 1-octadecene (7 mL) was added a water solution (2 mL) containing $Y(CH_3COO)_3$, $Gd(CH_3COO)_3$, $Yb(CH_3COO)_3$, and $Er(CH_3COO)_3$ with a total lanthanide content of 0.4 mmol. The resulting mixture was heated at 150 °C for 1 h to form lanthanide oleate complexes and then cooled to room temperature. Subsequently, a methanol solution (6 mL) containing NH_4F (1.6 mmol) and NaOH (1 mmol) was added and stirred at 50 °C for 30 min. The reaction temperature was then increased to 100 °C to remove the methanol from the reaction mixture. Upon removal of methanol, the solution was heated to 290 °C and maintained at this temperature under an argon flow for 1.5 h, at which time the mixture was cooled to room temperature. The resulting nanoparticles were

precipitated out through an addition of ethanol, collected by centrifugation, washed with ethanol, and finally redispersed in 4 mL of cyclohexane.

***In situ* growth of MnO_2 on the surface of hydrophobic UCNPs (UCNPs@MnO_2).**

Experimental group: A mixture of as-prepared UCNPs (0.1 g), cyclohexane (100 mL), *tert*-butanol (70 mL), water (10 mL) and 5 wt % K_2CO_3 aqueous solution (5 mL) was stirred at room temperature for about 20 min. Then 20 mL of Lemieux-von Rudloff reagent (17.1 mM $KMnO_4$ and 0.035 M $NaIO_4$ aqueous solution) was added dropwise, and the resulting mixture was stirred at about 40 °C for the desired time. The product was then isolated by centrifugation, washed with deionized water and ethanol, and dried under vacuum.

Control group 1: A mixture of as-prepared UCNPs (0.1 g), cyclohexane (100 mL), *tert*-butanol (70 mL), water (10 mL) and 5 wt % K_2CO_3 aqueous solution (5 mL) was stirred at room temperature for about 20 min. Then 20 mL of Lemieux-von Rudloff reagent (5.7 mM $KMnO_4$ and 0.105 M $NaIO_4$ aqueous solution) was added dropwise, and the resulting mixture was stirred at about 40 °C for the desired time. The product was then isolated by centrifugation, washed with deionized water and ethanol, and dried under vacuum.

Control group 2: A mixture of as-prepared UCNPs (0.1 g), cyclohexane (100 mL), *tert*-butanol (70 mL), water (10 mL) and 5 wt % K_2CO_3 aqueous solution (5 mL) was stirred at room temperature for about 20 min. Then 20 mL of Lemieux-von Rudloff reagent (17.1 mM $KMnO_4$ and 0.105 M $NaIO_4$ aqueous solution) was added dropwise, and the resulting mixture was stirred at about 40 °C for the desired time. The product was then isolated by centrifugation, washed with deionized water and ethanol, and dried under vacuum.

Control group 3: A mixture of cyclohexane (100 mL), *tert*-butanol (70 mL), water (10 mL) and 5 wt % K_2CO_3 aqueous solution (5 mL) was stirred at room temperature for about 20 min. Then 20 mL of Lemieux-von Rudloff reagent (17.1 mM $KMnO_4$ and 0.035 M $NaIO_4$ aqueous solution) was added dropwise, and the resulting mixture was stirred at about 40 °C for the desired time. The product was then isolated by

centrifugation, washed with deionized water and ethanol, and dried under vacuum.

Note: The concentrations of MnO_2 on the surfaces of UCNPs were quantified using inductively coupled plasma optical emission spectrometry (Optima 8000 ICP-OES spectrometer, Perkin-Elmer). The samples isolated at different oxidation time were collected and washed 5 times with ultrapure water, finally dried under vacuum and weighed. Then the obtained samples were dispersed in ultrapure water and divided into three equal parts (1,2,3). Sample 1 was used to determine the UV-vis absorption using a UV-2450 UV-vis spectrophotometer (Shimadzu). Sample 2 was used to test the quench response by an F4500 spectrometer (Hitachi) in conjuction with a 980 nm diode laser. Then, the fluorescence recovery was determined after adding 10 mM GSH. Sample 3 was treated with 10 mM GSH in ultrapure water for 2 h to reduce MnO_2 into Mn^{2+}, and was then divided into two equal parts (3a, 3b). Sample 3a was used to determine the concentration of Mn^{2+} using ICP-OES. Sample 3b was centrifuged and washed 5 times with ultrapure water to obtain the oxidized UCNPs only, and dried under vacuum. Finally the UCNPs were weighed, so the amount of MnO_2 growed on the surface of UCNPs could be calculated.

Half-quench concentration of MnO_2 toward to the fluorescence of UCNPs. We repeated the experiment 3 times according to the procedure of the experimental group in the supporting information. A serious of oxidized UCNPs at different oxidation time were collected to do fluorescence test and do ICP-OES test to achieve the concentration of Mn^{2+}, so a serious of pure weight of MnO_2 formed on the UCNPs could be calculated. Then GraphPad Prism software was applied to obtain the half-quench concentration of MnO_2 toward to the fluorescence of UCNPs.

Response of UCNPs@MnO_2 nanoparticles as MRI contrast agents in normal human whole blood. Whole blood was collected from **a** normal human, and was kept in an anticoagulative tube. The T1 relaxation times of UCNPs@MnO_2 (The weight ratio of MnO_2/UCNPs was 294.64 μg/mg) nanoparticles at different concentrations (0, 10, 25, 50, 100, 200, 400 μg/mL) treated with the fresh whole blood were measured at 1.5 T on a Bruker Minispec analyzer (1.5T).

Preparation of mesoporous silica-coated UCNPs@MnO_2 nanoparticles (UCNPs@MnO_2@$mSiO_2$). 0.5 g of CTAC, 0.02 g of TEA and 20 L of water were mixed and stirred at 80 °C until a transparent emulsion was formed. Then 10 mL of the as-prepared UCNPs@MnO_2 (The weight ratio of MnO_2/UCNPs was 294.64 μg/mg) dispersed in water was added and stirred for 1 h. Next, 0.04 mL TEOS was added dropwise into the solution, followed by stirring for 12 h. UCNPs@MnO_2@$mSiO_2$ nanoparticles were washed with ethanol/water (1:1 v/v) twice and redispersed in a 40 mL NaCl (1%) solution to remove CTAC templates. The extraction was repeated three times, and the products were washed with deionized water.

Preparation of MnO_2 nanosheets. A-20 mL mixed aqueous solution of 0.6 M tetramethylammonium hydroxide and 3 wt % H_2O_2 was added to 10 mL 0.3 M $MnCl_2$ solution within 15 s. The solution became dark brown immediately as the two solutions were mixed. The resulting dark brown suspension was stirred vigorously overnight in the open air at room temperature. The as-prepared bulk manganese dioxide was centrifuged at 2000 rpm for 10 minutes and washed with copious amounts of distilled water and methanol. Then, the bulk manganese dioxide was dispersed in 20 mL of water and ultrasonicated for 10 h. Then the dispersion was centrifuged at 2000 rpm, and the supernatant was kept for further use. The concentration of as-prepared MnO_2 nanosheets (0.05 mg/mL) was quantified by inductively coupled plasma optical emission spectrometry (Optima 8000 ICP-OES spectrometer, Perkin-Elmer).

Doxorubicin loading and gelatin capping (Dox-loaded nanosystem). 1 mg of UCNPs@MnO_2@$mSiO_2$ nanosystem was mixed with 0.5 mg of Dox in a 2-mL mixture of DMF and H_2O (1:1). After shaking for 24 h at RT in the dark, the Dox-loaded UCNPs@MnO_2@$mSiO_2$ were collected by centrifugation. The residual particles were gently shaken with an aqueous gelatin solution (1 mL, 1%) at 50 °C for 6 h to achieve pore saturation. Then, deionized water (8 mL) at 4 °C was quickly poured into the mixture. After two cycles of centrifugation/water rinsing/redispersion,

50 µL of a 1% glutaraldehyde solution was added to crosslink the gelatin at 4 °C. The crosslinking reaction was continued for 8 h. Then, the Dox-loaded nanosystem samples were centrifuged, rinsed with water, and redispersed three times. To evaluate Dox-loading capacity, the supernatant and the washing solution were collected. Dox content in the supernatant solution before and after incubation was measured by a UV-2450 UV-vis spectrophotometer. The drug-loading efficiency was measured to be approximately 0.042 mmol/g.

DNA preparation. All DNA synthesis reagents were purchased from Glen Research (Sterling, VA), and all DNA probes (see sequences in Table S1) were synthesized on an ABI3400 DNA/RNA synthesizer (Applied Biosystems, Foster City, CA, USA) based on solid-state phosphoramidite chemistry at a 1 µmol scale. An amino or phosphate group was coupled on the 5'- ends of primers and templates (see Table S1 for sequences), if applicable. DNA sequences were deprotected according to the manufacturer's instructions. Deprotected DNA was further purified by reversed-phase HPLC (ProStar, Varian, Walnut Creek, CA, USA) on a C-18 column using 0.1 M triethylamine acetate (TEAA, Glen Research Corp.) and acetonitrile (Sigma Aldrich, St. Louis, MO) as the eluent. The collected DNA products were dried and detritylated by dissolving and incubating DNA products in 200 µL 80% acetic acid for 20 minutes. The detritylated DNA product was precipitated with NaCl (3 M, 25 µL) and ethanol (600 µL). UV-Vis measurements were performed with a Cary Bio-100 UV/Vis spectrometer (Varian) for DNA quantification.

Sgc8 aptamer-labeling of Dox-loaded nanosystem (sgc8-nanosystem-Dox). Excess Sulfo-SMCC (4 mg) was added to a mixture solution of 1×PBS (pH 7.4) and DMF (7:3 v/v). Then Dox-loaded nanosystem (10 mg) were suspended in the above solution for 2 hours. The resulting particles were collected by centrifugation and extensively washed with 1×PBS (pH 7.4) for 3 times. Further functionalization of the thiol-sgc8 aptamer was performed by mixing excess sgc8 aptamer (20 nM) with the maleimide-modified nanosystem in 1×PBS (pH 8.0) and shaking at room temperature

for 24 h. The particles were recovered by centrifugation and washed with 1×PBS (pH 7.4).

Dynamic Light Scattering (DLS) of zeta potential test and size test. DLS measurements were obtained using a Malvern Instruments Zetasizer Nano ZS. Data plots and standard deviations were calculated from an average of three measurements, each of which consisted of 10 runs of 45 seconds. Samples for zeta potential test were suspended in 1×PBS buffer, pH 7.4 or pH 5.0. Samples for size test were suspended in water.

Agarose gel electrophoresis test. UCNPs@MnO_2@$mSiO_2$@gel nanosystem (0.5 mg/mL), sgc8 (2 μM) and sgc8-nanosystem (2 μM) were all prepared in 1×PBS. Electrophoresis was carried out in 1×Tris-borate-EDTA (TBE) buffer (90 mM boric acid, 10 mM EDTA, pH 8.0) at 100 V for 30 min. The sample concentration of each well was 10 μM. The 2.5% agarose gel was stained with ethidium bromide in advance.

***In vitro* Dox release test.** Dox release experiments were performed in 1×PBS at pH values of 4.0, 5.0, 6.0, and 7.4, respectively. Sgc8-nanosystem-Dox nanoparticles (2 mg/mL) were dispersed in 1×PBS with various pH values. These samples were shaken at 100 rpm at 37 °C. The release profiles of DOX molecules from the pores to aqueous solution were monitored via the fluorescence intensity of the dye centered at 560 nm by a Fluoromax-4 spectrofluorometer (Horiba Scientific, Edison, NJ).

Cell-surface binding assays using flow cytometry. For the binding analysis, CEM or Ramos cells with a concentration of 10^6 cells/mL were incubated with 50 μg/mL of sgc8-nanosystem or lib- nanosystem on ice in a 200 μL volume of binding buffer for 30 min. The cells were centrifuged, washed three times with 200 μL of washing buffer, redispersed in 200 μL of binding buffer, and subjected to BD FACSVerse™ flow cytometry analysis by counting 10000 events. The data were analyzed with FlowJo software. FITC-labeled sgc8 sequence or library sequence provided the fluorescence signal.

Confocal laser-scanning microscopy imaging. For the internalization test, the sgc8-nanosystem (50 μg/mL) were incubated with CEM and Ramos cells ($1*10^6$ cells/mL) at 4 °C for 0.5 h, followed by washing with washing buffer to remove the unbound samples, then fresh medium with 2% FBS was added to incubate for another 4 h, and then wash the sample with washing buffer. The cells were suspended in washing buffer and analyzed using an FV1000-MPE multiphoton laser scanning confocal microscope (Olympus). The signal was due to the recovered fluorescence of UCNPs. 980 nm laser was used as excitation light source. For Time scanning of confocal imaging for HeLa cells, HeLa Cells were plated in a 35 mm confocal dish (coverglass-bottom dish) (MatTek Corp., Ashland, MA, USA) and grown to around 80% confluence for 24 h before the experiment. Cells were washed twice with washing buffer, and then incubated with sgc8-nanosystem (50 μg/mL) in binding buffer at 4 °C in 5% CO_2, followed by washing twice with washing buffer, then binding buffer was added to incubate for another 5 h. All cellular fluorescence images at different incubation time were collected on an FV1000-MPE multiphoton laser scanning confocal microscope (Olympus, Japan). The signal was due to the recovered fluorescence of UCNPs. A 980 nm laser was the excitation source for UCNPs.

For colocalization test with lysosome, LysoTracker Green (Life Technonogy, Beijing, China) with a standard concentration was added into HeLa cells and incubated at 37 °C for 30 min. The LysoTracker was removed by washing and ready-to-use. A 60x oil-immersion objective on FV1000-MPE multiphoton laser scanning confocal microscope (Olympus, Japan) was used for imaging.

Response of sgc8-nanosystem-Dox nanoparticles as MRI contrast agents to GSH at pH 5.0 and pH 7.4. First, sgc8-nanosystem-Dox nanoparticles ([Mn^{2+}]= 0.00, 0.14, 0.71, 1.42, 2.14, 2.85 mM, tested by ICP-OES) (The weight ratio of MnO_2/UCNPs was 294.64 μg/mg) were dispersed in PBS with pH 5.0 and pH 7.4 for 5 h to dissolve the gelatin layer. Then the T_1-weighted signals of sgc8-nanosystem-Dox at different concentrations ([Mn^{2+}]= 0.00, 0.14, 0.71, 1.42, 2.14, 2.85 mM, tested by ICP-OES) treated with or without 10 mM GSH were measured at 1.5 T on a Bruker Minispec analyzer (1.5 T). The T_1-weighted of samples mentioned above were acquired on a

Signa HDXT 3.0 T instrument (GE Healthcare) with a gradient echo sequence (TR = 2400 ms and TE = 63.0 ms).

***In vitro* MRI of cells.** CEM or Ramos cells (1x10^7 cells) were incubated with sgc8-nanosystem (The weight ratio of MnO_2/UCNPs was 294.64 μg/mg) at concentration of 0, 25 and 50 μg/mL at 37 °C for 3 h in a volume of 2 mL medium without FBS. At the predetermined time, cells were washed twice with DPBS after the supernatant was removed. Then, cells were mixed with 0.5% agarose. The mixture was transferred to a 96-well plate for MRI. T_1-weighted MRI of the cells mixed with agarose was performed with a Signa HDXT 3.0 T instrument. T_1-weighted MRI images were acquired with a gradient echo sequence (TR = 480 ms and TE = 10.0 ms).

Measurement of intracellular manganese content. CEM cells and Ramos cells (10^7 cells) were incubated with sgc8-nanosystem (The weight ratio of MnO_2/UCNPs was 294.64 μg/mg) at concentration of 50 μg/mL in a volume of 2 mL buffer for 4 h at 37 °C. After removing the supernatant, cells were washed twice with DPBS. Then the cells or MRI cellular sample were treated with ultrapure water. After cell lysate, the broken cell fragments were removed using a 0.28-μm filter, the volume of these samples was adjusted to 3 mL. The measurement of manganese content was carried out on ICP-OES. The cellular manganese concentration was calculated by dividing the total manganese content by the number of cells.

Cytotoxicity study. A sample of 1×10^3 cells in 50 μL of washing buffer was seeded into each test well on a 96-well plate. Then a series of samples at the desired concentration in 50μL of washing buffer were added to the respective test well. The cells were incubated at 37 °C in a 5% CO_2 atmosphere for 2 h. Then, the supernatant was removed from the test well after centrifugation and 100 μL of fresh cell culture medium was added. After another 48h of incubation at 37 °C in a 5% CO_2 atmosphere, a standard MTS assay was performed. The absorbance value at 570 nm was determined by a microplate reader.

Table S1. DNA sequences used in this work

Sequences	5'-3'
Sgc8	FAM-ATC TAA CTG CTG CGC CGC CGG GAA AAT ACT GTA CGG TTA GAT TTT TT-SH
lib	FAM-TTT TTT TTT TTT TTT TTT TTT TTT TTT TTT TTT TTT TTT TTT TTT TTT TT-SH

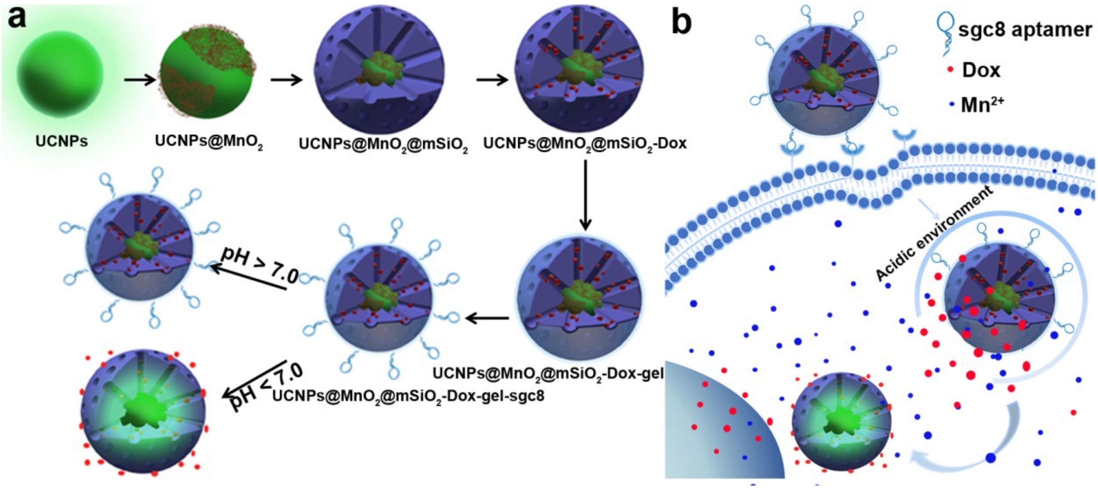

Scheme S1. a) Each fabrication step of the nanosystem. b) The working priciple for pH-responsive controlled release of the UCNPs@MnO_2@$mSiO_2$-Dox@gel-sgc8 nanosystem (sgc8-nanosystem-Dox).

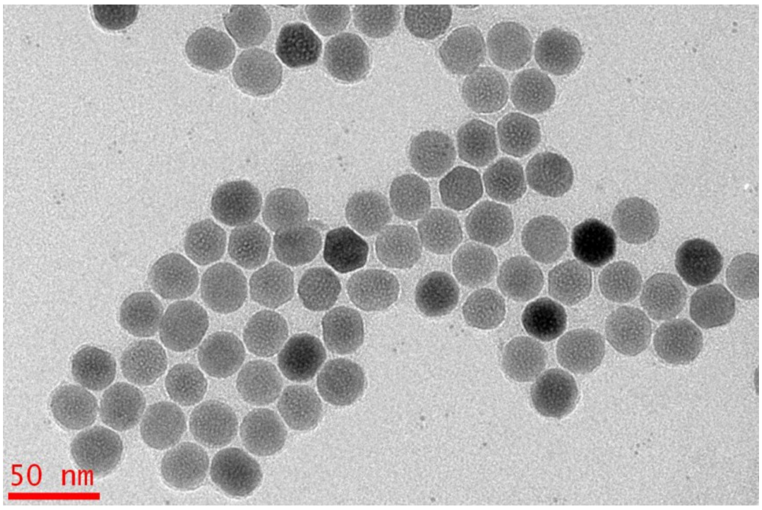

Figure S1 TEM characterization of UCNPs, demonstrating their monodisperse particle size of about 22 nm. The UCNPs were dispersed in cyclohexane.

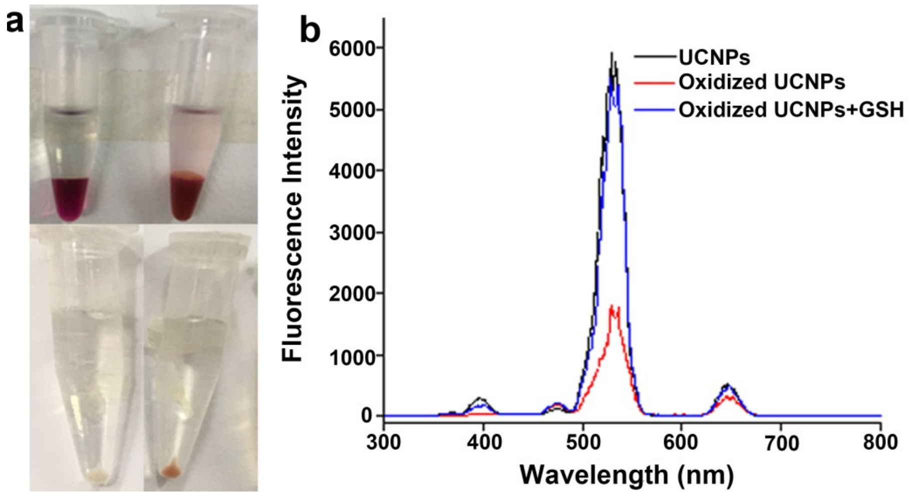

Figure S2 a) Pictures of UCNPs at 0 h (left) and the oxidized UCNPs after 60 h (Right). The upper picture shows the heterogeneous reaction products. The lower picture shows the heterogeneous reaction products after washing with water and centrifugation. b) Fluorescence quenching and recovery of UCNPs and the oxidized UCNPs. 74.6% of the upconverted luminescence of the UCNPs was quenched after 60 h oxidization, and the fluorescence recovered after adding GSH. It can be deduced from these results that something formed on the surfaces of UCNPs.

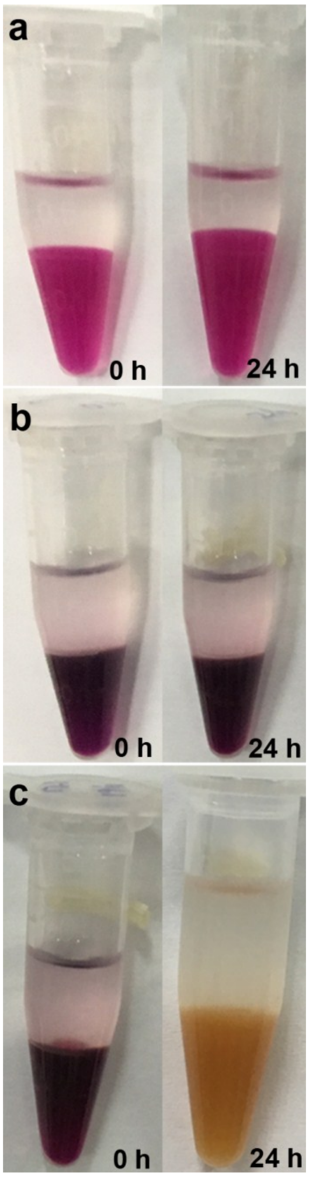

Figure S3 Pictures of the reaction mixture at different times (0 h and 24 h). (a) Control group 1; (b) Control group 2; (c) Experimental group. The results showed the color change only under conditions of increased permanganate concentration and decreased periodate concentration.

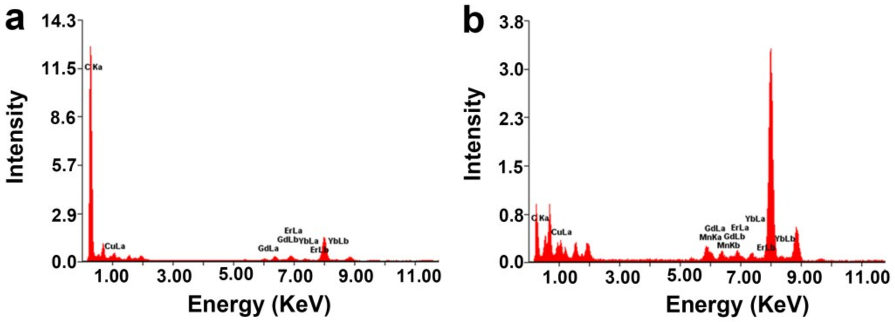

Figure S4. Energy-dispersive X-ray (EDX) analysis of a) UCNPs, and b) oxidized UCNPs (24 h). In the spectra of the oxidized UCNPs, new element of Mn was observed.

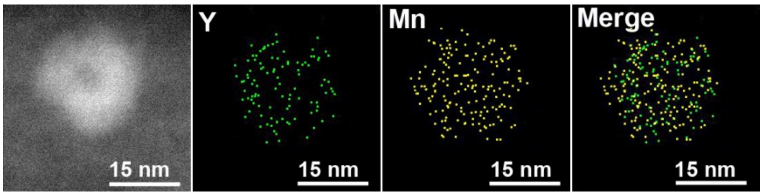

Figure S5. STEM images of UCNPs@MnO_2 (24 h) nanostructures and the elemental mapping. From left to right: dark field, Y mapping, Mn mapping, and merge of Y and Mn.

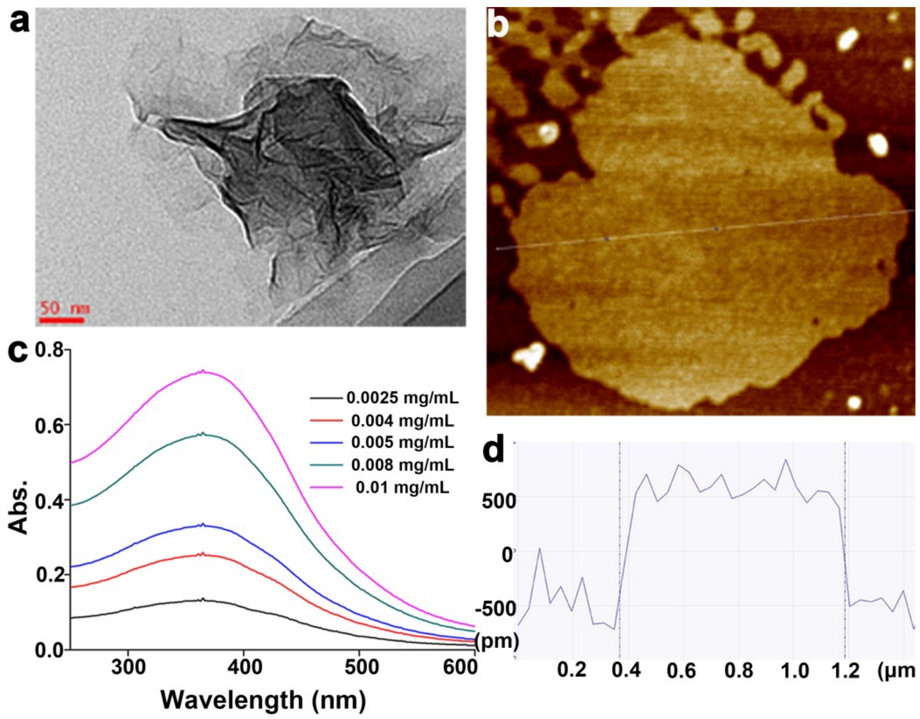

Figure S6. TEM (a), AFM (b and d), and UV (c) characterization of the positive control of MnO_2 nanosheets. The black line in (d) represents the height profile of the section in (b) labeled with a line. From Figure 3c, it can be concluded that the absorption of MnO_2 increased with the concentration of MnO_2 nanosheets.

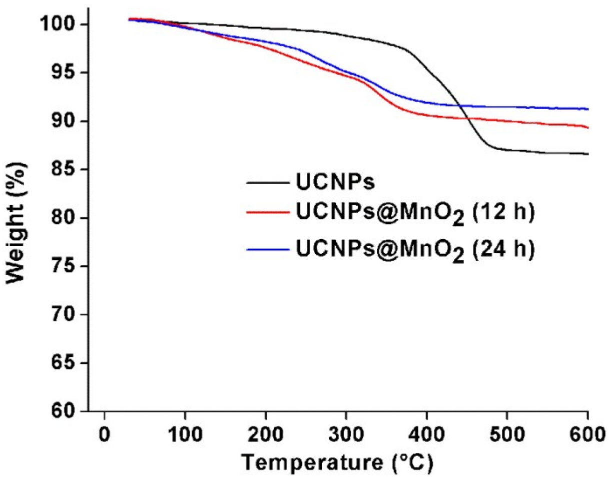

Figure S7. The TG curve of UCNPs and the oxidized UCNPs after 12 h and 24 h. TG curves were measured using a STA449C thermogravimetric analyzer from room temperature to 600 °C with heating rate of 10 °C/min and an argon flow rate of 60 mL/min. The oxidized UCNPs@MnO_2 samples were treated with GSH to remove the effect from the surface MnO_2.

Calculation Parts:

The molecular weight of oleic acid (OA) and azelaic acid (AA):

$$M_{OA}=282.46 \text{ g/mol}, M_{AA}=188.22 \text{ g/mol}$$

(1) UCNPs@MnO_2 (24 h)

When the content of OA was: $wt\%_{OA}=10.5\%$,

so theoretically :

$$wt\%_{AA} = wt\%_{OA} - \frac{M_{OA}-M_{AA}}{M_{OA}} \times 100\%$$
$$= 10.5\% - \frac{282.46-188.22}{282.46} \times 100\%$$
$$= 7.2\%$$

(2) UCNPs@MnO_2 (12 h)

From Figure S6, the ligand content on the surface of UCNPs@MnO_2 was 8.7%, which was OA and AA, so $wt_{OA+AA}\%=8.7\%$

Hypothesis that the content of OA was x%: $wt\%_{OA}=10.5\%$

So, $W_{AA} = (8.7-x)\%$

According to the equation:

$$\frac{M_{OA} * x\%-M_{AA} * (wt_{OA+AA}\%-x)\%}{M_{OA}} = wt\%_{OA}-wt\%_{OA+AA}$$
$$\frac{282.46*x\%-188.22*(8.7-x)\%}{282.46} = 10.5\% - 8.7\%$$
$$x = 4.6$$

So the oxidized ratio of OA (12 h) was:

$$wt\%_{OA'} = \frac{10.5\%-4.6\%}{10.5\%} \times 100\%$$
$$= 56.2\%$$

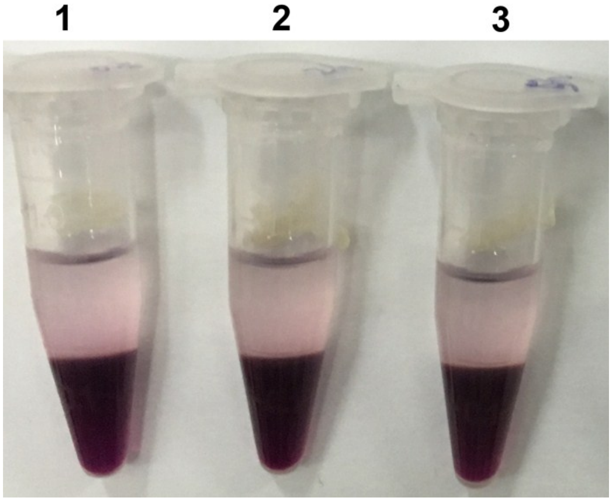

Figure S8. The reaction mixtures of the control experiment in the absence of oleic acid-capped UCNPs. 1: 0 h; 2: After one week; 3: 2 after centrifugation. The results showed that no change in the color of the reaction mixture occurred after reacting for one week, and no new product was produced after centrifugation, indicating that $KMnO_4$ and $NaIO_4$ solution by itself is quite stable under the experimental condition. These results show the specificity towards olefinic bonds of this oxidation reagent and that the reagents alone could not bring about the growth of MnO_2.

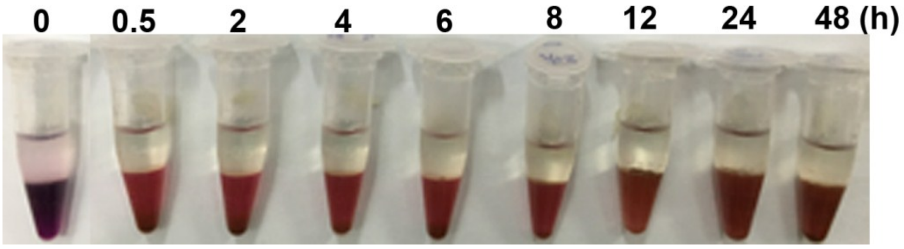

Figure S9. Pictures of the collected supernatants after centrifugation and being putting for another 3 days. It can be observed that the colors of all the supernatants changed after 3 days, indicating that the MnO_2 precipitate came from the reaction of intermediates formed during the oxidation process.

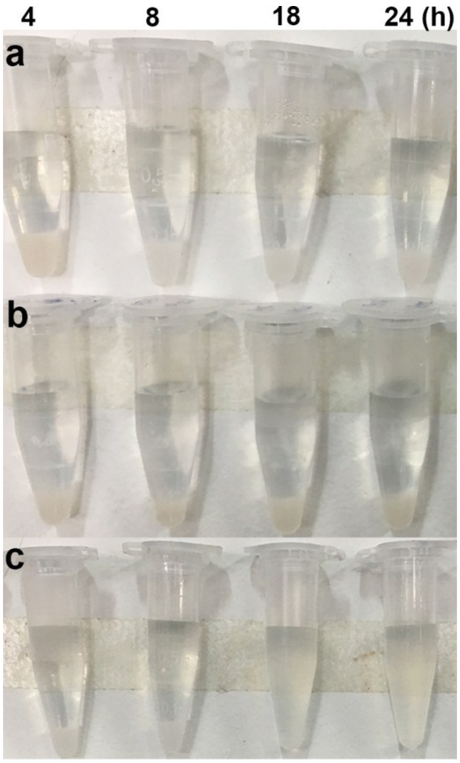

Figure S10. The dispersibility of different time products treated with GSH in PBS. (a) Control group 1; (b) Control group 2; (c) Experimental group. The aggregates were observed soon in control group 1 and 2 after adding GSH, indicating the poor dispersibility of the products after 24-h oxidation. However, in experimental group, the dispersibility was enhanced a lot after 18-h oxidation when the products were put for overnight.

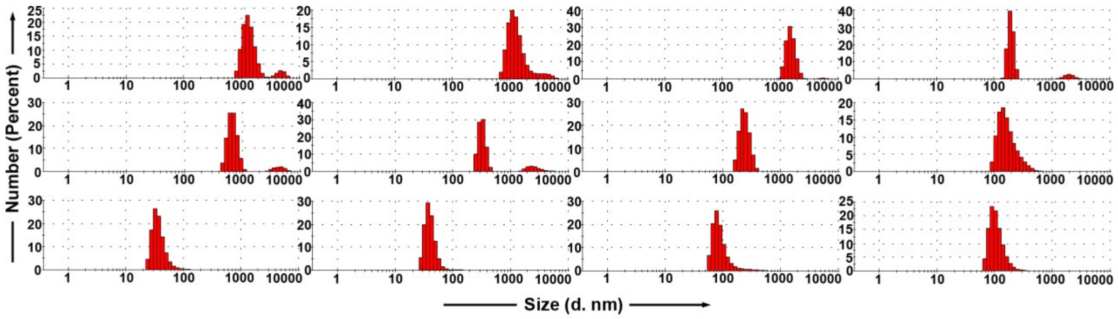

Figure S11. DLS data of oxidized UCNPs at different time without treatment with GSH. Top: Control group 1; Middle: Control group 2; Bottom: Experimental group. Form left to right, the oxidation time was 4 h, 8 h, 18 h, and 24 h, respectively. The results further indicate that the growth of MnO_2 on the surfaces of UCNPs improved the dispersibility of UCNPs in the experimental group. However, the dispersibility was poor, or otherwise, the oxidized UCNPs were aggregated into large particles in the two control groups, correlating nicely with the results in Figure 1. Again, these DLS results indicate that our method increases the rate of the entire oxidation process.

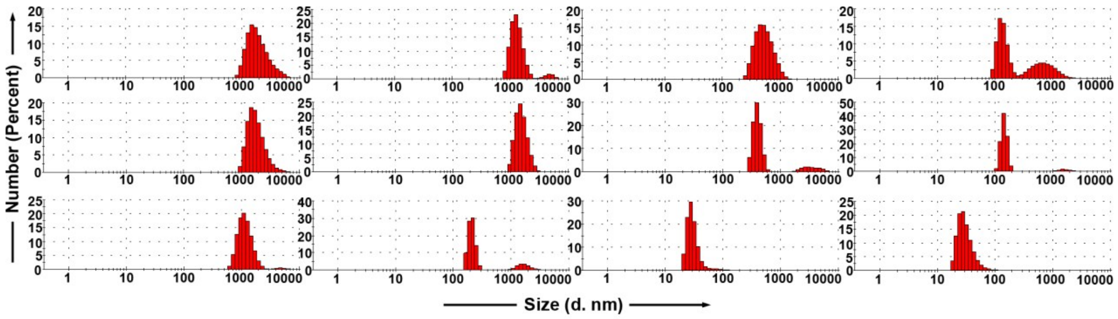

Figure S12. DLS data of oxidized UCNPs at different time with treatment with GSH (10 mM). Top: Control group 1; Middle: Control group 2; Bottom: Experimental group. Form left to right, the oxidation time was 4 h, 8 h, 18 h, and 24 h, respectively. The samples, which were the same as the samples used for DLS test in Figure S11, were treated with 10 mM GSH to reduce the MnO_2 grown on the surfaces of UCNPs. We can see that the dispersibilities and stabilities of the oxidized UCNPs (after 18 h) in the experimental group were improved considerably, but the dispersibility of the two control groups was poor because of the incomplete oxidation of OA ligands, correlating nicely with the results in Figure S10. Again, these DLS results indicate that our method increases the rate of the entire oxidation process.

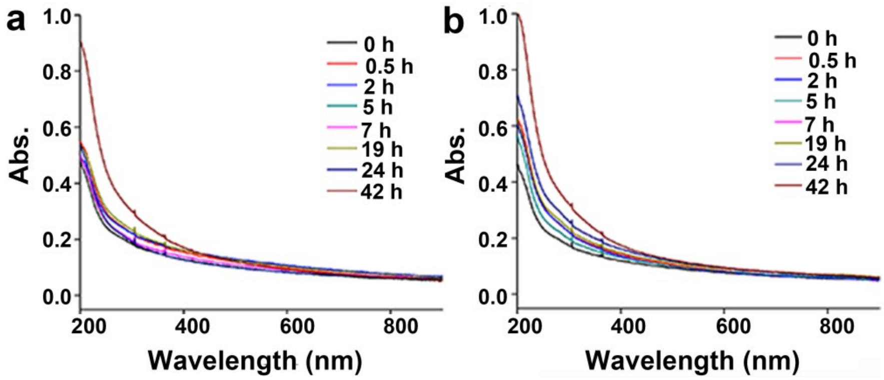

Figure S13. The UV-vis absorption spectra of control group 1 (a) and control group 2 (b). The results did not show the characteristic broad peak centered at 380 nm for MnO_2 nanomaterials compared with the UV-vis absorption spectroscopy of the experimental group (Figure 4b), indicating that our improved method accelerated the formation of MnO_2 on the surfaces of the oxidized UCNPs.

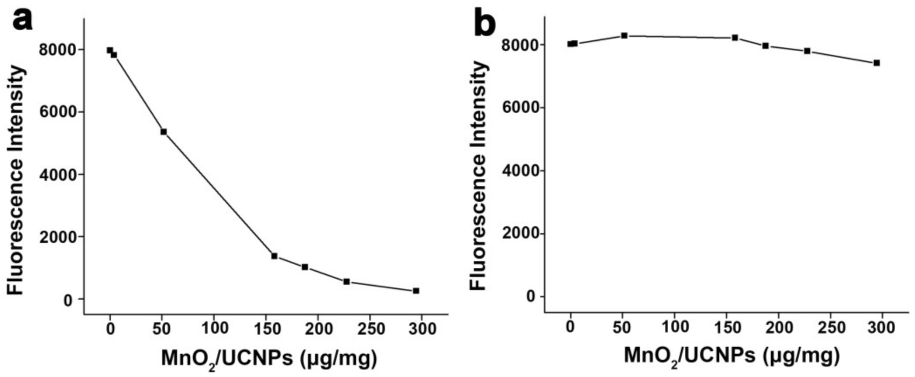

Figure S14. Plots of (a) fluorescence intensity at 533 nm versus different MnO_2/UCNPs concentration ratios (0-294.64 μg/mg), and (b) Fluorescence recovery response of the samples in (a) after adding 10 mM GSH.

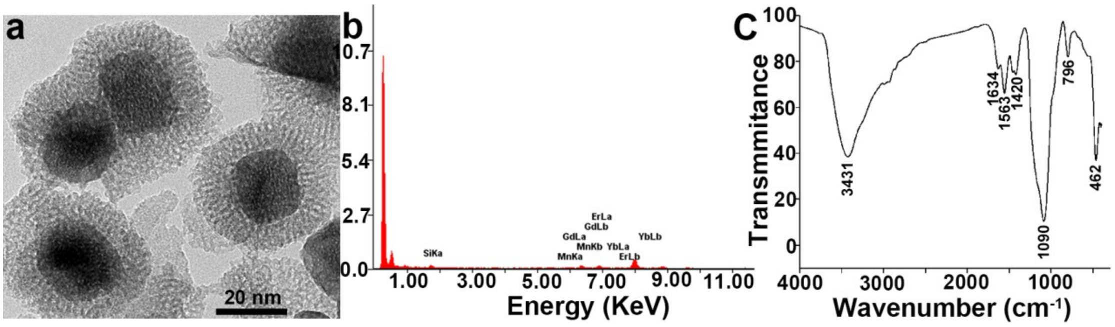

Figure S15. Characterization of UCNPs@MnO_2@$mSiO_2$. (a)TEM image, (b) EDX result indicating the presence of Si in the sample, and (c) FT-IR spectra. A new strong absorption peak at 1090 cm^{-1}, which, in comparison with Figure3d, corresponded to the Si-O-Si asymmetric stretching vibration. These results demonstrated the successful synthesis of UCNPs@MnO_2@$mSiO_2$.

Gelatin, a natural proteinaceous biopolymer, has a long history of safe and wide application with unique properties of natural origin, low- cost, low- toxicity, pH-biodegradability, nonimmunogenicity, and flexible chemical modification because of its many carboxyl, amine, and amide functional groups on the peptide residues.

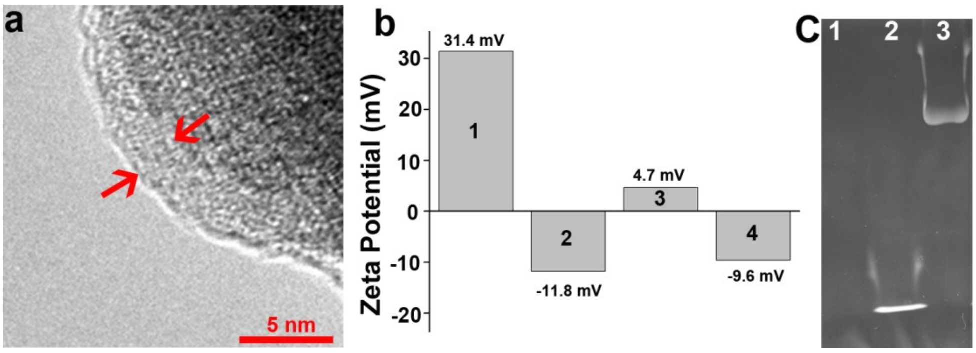

Figure S16. (a) TEM characterization of gelatin capping of the nanosystem. (b) Zeta potential test of the product of each step (pH 7.4). 1: UCNPs@MnO_2; 2: UCNPs@MnO_2@mSiO_2; 3: UCNPs@MnO_2@mSiO_2@gel; 4: UCNPs@MnO_2@mSiO_2@gel-sgc8 (sgc8-nanosystem). After conjugating with sgc8 aptamer, the zeta potential is more negative, demonstrating the successful conjugation of sgc8 aptamer on the surface of gelatin. (c) Agarose gel electrophoresis tests of UCNPs@MnO_2@mSiO_2-gel nanosystem (Lane 1), sgc8 (Lane 2) and sgc8-nanosystem (Lane 3). The agarose gel electrophoresis test showed no signals for UCNPs@MnO_2@mSiO_2-gel nanosystem as expected (lane 1). Lane 3 clearly showed that the electrophoretic mobility of sgc8-nanosystem was much slower than that of the sgc8 (lane 2), indicating that sgc8 aptamers were successfully conjugated on UCNPs@MnO_2@mSiO_2-gel nanosystem. The agarose gel concentration was 2.5%, and run at 100 V for 30 min. The gel was prestained by ethidium bromide.

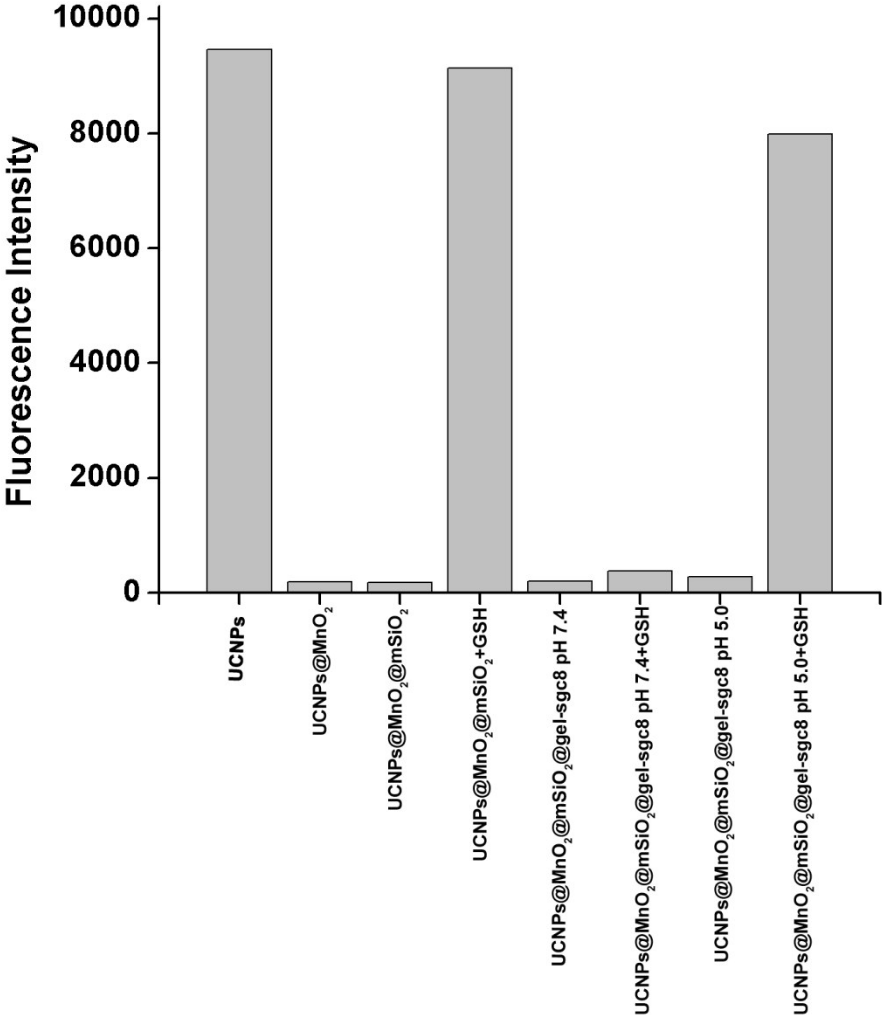

Figure S17. Histogram of different assembled nanoparticles at different condition versus their corresponding fluorescence intensity.

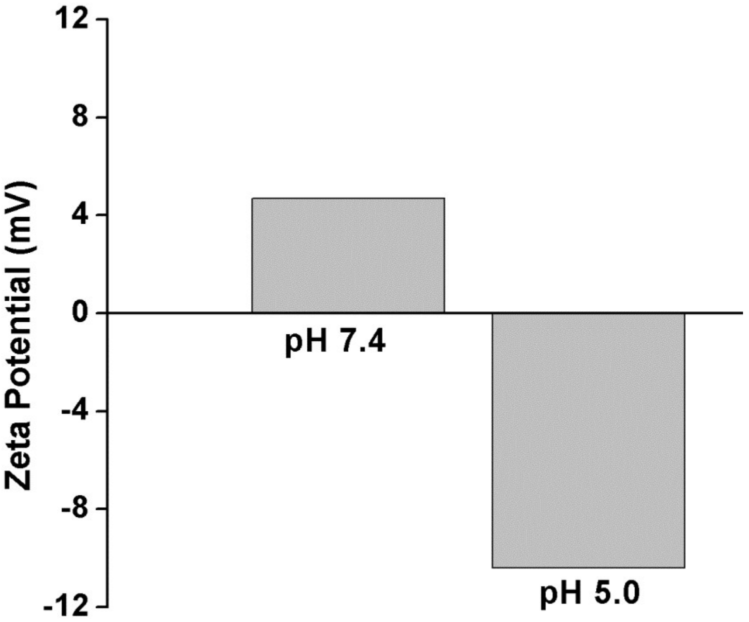

Figure S18. Zeta potential of UCNPs@MnO_2@$mSiO_2$@gel at different pH. After crosslinking, the charge of the cross-linked gelatin layer was negative (-10.4 mV) in the acidic environment (pH 5.0), and the charge of mesoporous silica was negative (-11.8 mV), too. Hence, the increased electrostatic repulsive force between the gelatin coating layer and mesoporous silica could open the pore and allow the escape of the drug and the entrapped GSH molecules. However, in the neutral pH, the charge of the cross-linked gelatin was positive (4.7 mV), the electrostatic attraction force between the gelatin coating layer and mesoporous silica could stabilize the pore-blocking capability of the gelatin layer.

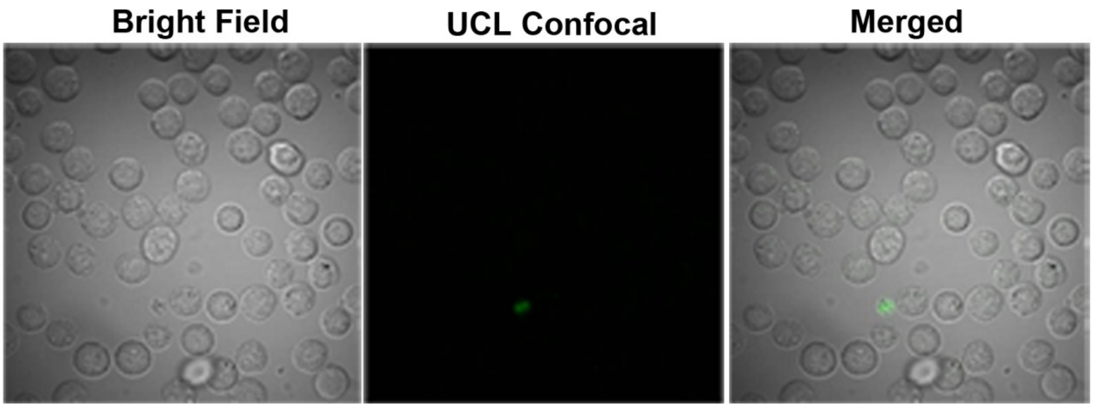

Figure S19. Confocal images of Ramos cells incubated with sgc8-nanosystem. The green fluorescence is due to UCNPs. 980 nm laser was used as excitation light source. Ramos cells incubated with sgc8-nanosystem for 4 h exhibited much less internalization ability compared with CEM cells (Figure 6c).

Time scanning confocal imaging of HeLa cells was performed to study time-dependent recovery of UCNPs fluorscence.

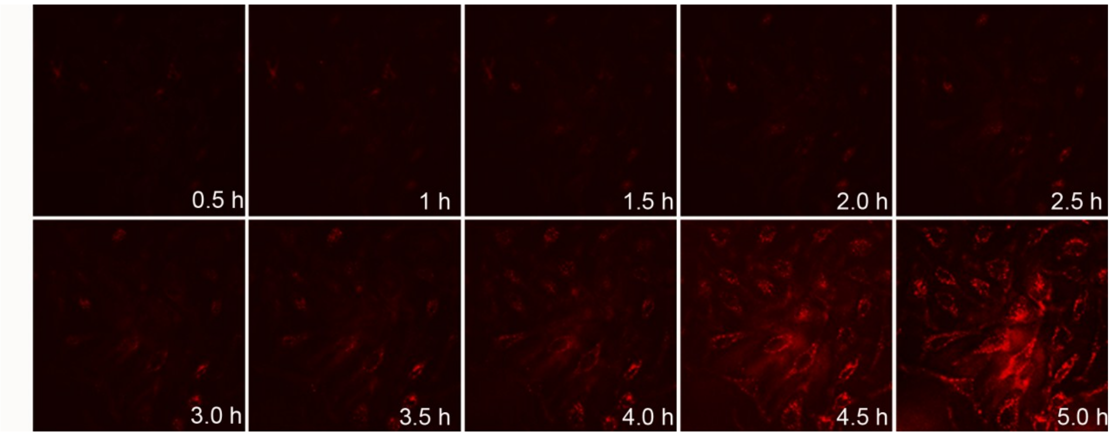

Figure S20. Time scanning of confocal imaging for HeLa cells incubated with sgc8-nanosystem. The fluorescence is due to the recovered fluorescence of UCNPs. 980 nm laser was used as excitation light source. The fluorescence of UCNPs became stronger and stronger with increasing incubation time, indicating time-dependent recovery of UCNPs fluorscence, which is due to time-dependent dissolution of the gelatin-coated layer. These results demonstrated that our sgc8-nanosystem could be used in bioimaging as a target-activatable luminescent probe for live cells.

To investigate the acid-induced and controlled-release property, the sgc8-nanosystem-Dox was exposed to different buffers with pH values of 4.0, 5.0, 6.0 and 7.4. The precise control of drug release was demonstrated by monitoring the concentration of the released anticancer drug by the measurement of fluorescence intensity at 560 nm (λ_{ex} = 488 nm).

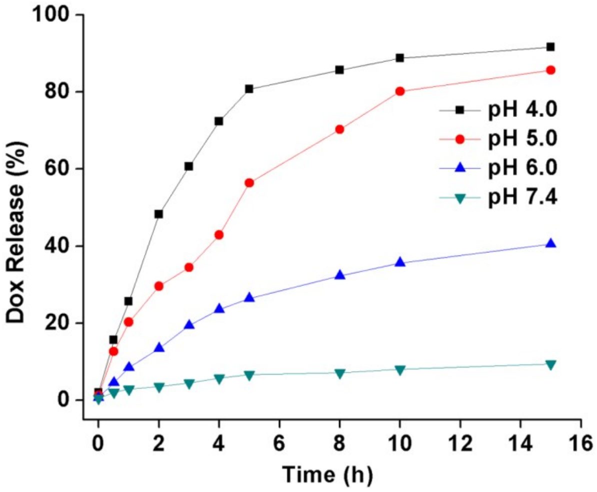

Figure S21. Release profiles of Dox from the sgc8-nanosystem-Dox at different pH values. The amount of Dox released from the nanosystem was less than 3.6 % after 5 h at neutral pH, indicating that the gelatin coating on UCNPs@MnO_2@$mSiO_2$ exhibits good storage and sealant effects in physiological solution (pH7.4). In contrast, increased acidity resulted in a significant release of Dox within 5 h at pH 6.0, 5.0, and 4.0, respectively, indicating that the release of Dox from the as-prepared nanoplatform is both time- and pH-dependent. As such, these findings revealed that the release rate of the pH-responsive sgc8-nanosystem-Dox increased over the 5 h period in mimicked environments of late endosomes and lysosomes, where the pH values would be in the range of 5.0-6.0.

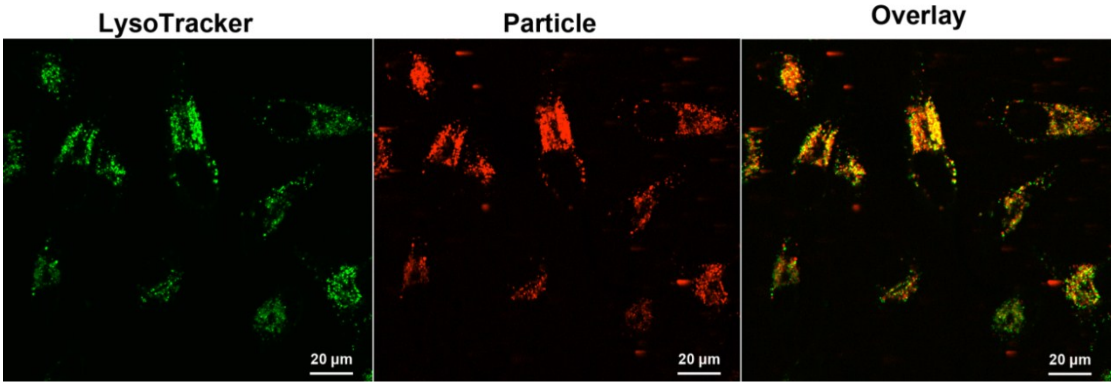

Figure S22. Colocalization study of sgc8-nanosystem (red) with LysoTracker Green (green) in HeLa cells. LysoTracker Green was used to stain the cell acidic organelles-lysosomes. The red signal was from UCNPs with exciting light of 980 nm laser. Cells were imaged using a 60 oil-immersion objective. Yellow signal were obtained after green signal merged with red signal, but there were still some red signal in the overlay picture, which, in some extent, confirmed that part of the particles had escaped from lysosomes within 2.5 h.

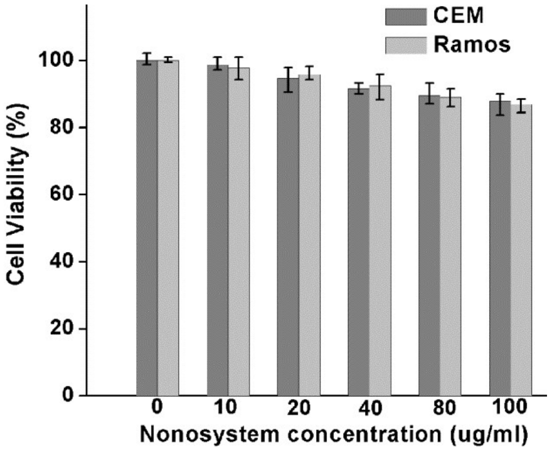

Figure S23. Cytotoxicity of sgc8-nanosystem on CEM and Ramos cells. The MTT results demonstrate that sgc8-nanosystem without Dox loading has almost no cytotoxicity to CEM and Ramos cells.

Reference

[1] L. S. Magee, *LabNotes*, 2005, **15**, 1–4.
